# A low-cost open-source 5-choice operant box system optimized for electrophysiology and optophysiology in mice

**DOI:** 10.1038/s41598-021-01717-1

**Published:** 2021-11-15

**Authors:** Sampath K. T. Kapanaiah, Bastiaan van der Veen, Daniel Strahnen, Thomas Akam, Dennis Kätzel

**Affiliations:** 1 Institute of Applied Physiology, Ulm University, Ulm, Germany; 2 Department of Experimental Psychology, University of Oxford, Oxford, UK

**Keywords:** Cognitive neuroscience, Learning and memory, Neural circuits

## Abstract

Operant boxes enable the application of complex behavioural paradigms to support circuit neuroscience and drug discovery research. However, commercial operant box systems are expensive and often not optimised for combining behaviour with neurophysiology. Here we introduce a fully open-source *Py*thon-based *o*perant-box *s*ystem in a *5*-choice design (pyOS-5) that enables assessment of multiple cognitive and affective functions. It is optimized for fast turn-over between animals, and for testing of tethered mice for simultaneous physiological recordings or optogenetic manipulation. For reward delivery, we developed peristaltic and syringe pumps based on a stepper motor and 3D-printed parts. Tasks are specified using a Python-based syntax implemented on custom-designed printed circuit boards that are commercially available at low cost. We developed an open-source graphical user interface (GUI) and task definition scripts to conduct assays assessing operant learning, attention, impulsivity, working memory, or cognitive flexibility, alleviating the need for programming skills of the end user. All behavioural events are recorded with millisecond resolution, and TTL-outputs and -inputs allow straightforward integration with physiological recordings and closed-loop manipulations. This combination of features realizes a cost-effective, nose-poke-based operant box system that allows reliable circuit-neuroscience experiments investigating correlates of cognition and emotion in large cohorts of subjects.

## Introduction

Operant boxes are an important tool for pre-clinical drug discovery and biological psychiatry, enabling the use of well-controlled psychological tests that have been adapted for rodents from their human counterparts^1,2^. However, commercial operant box systems have several disadvantages. High cost constrains the ability to scale up cohort sizes and hence the efficient use of experimenter time. Their mechanical design is often not optimised for neurophysiology, for example they often feature relatively deep recesses for poke holes and reward receptacles, which are poorly suited for testing implanted and tethered animals. Box designs are also rarely optimized for rapid turn-over of animals, due to the use of metal grid floors and layouts that take significant amount of time to clean after testing.

We here introduce an open source *Py*thon-based *o*perant-box *s*ystem that realizes a versatile *5*-choice layout (pyOS-5) and improves on current commercial systems, including reducing cost by ca. 80–85%, depending on the sourcing of individual parts. A 5-hole design was chosen because it allows the implementation of a wide variety of established tasks^2,12,13,19^ due to the large number of potential stimulus configurations and the low probability to obtain rewards by chance-level performance. Additionally, we designed a sound-insulating outer cubicle, peripheral circuit boards for operation, and low-cost peristaltic and syringe pumps for reward delivery. Complementing the hardware, we provide an open-source graphical user interface (GUI) specialised for running several tasks on the 5-choice setup and for automatically extracting commonly used behavioural metrics from the data. Within the emerging field of low-cost open-source operant box systems^3–6^ and software to control operant experiments^7–9^, our system is designed with a particular focus on the *end-user*, meeting five core demands: (1) Plug’n play set-up due to maximum integration of dedicated hardware and software components, (2) easy operation without requiring programming skills, (3) fast turn-over times between animals and direct extraction of both aggregated and millisecond time-resolved data, (4) optimal suitability of the box design, electronic interfaces, and software for simultaneous neurophysiological recordings and manipulations, and (5) low cost (well below < 1000 EUR per box) without any reliance on proprietary hardware or software.

## Methods

### Control electronics

**Figure 1:**
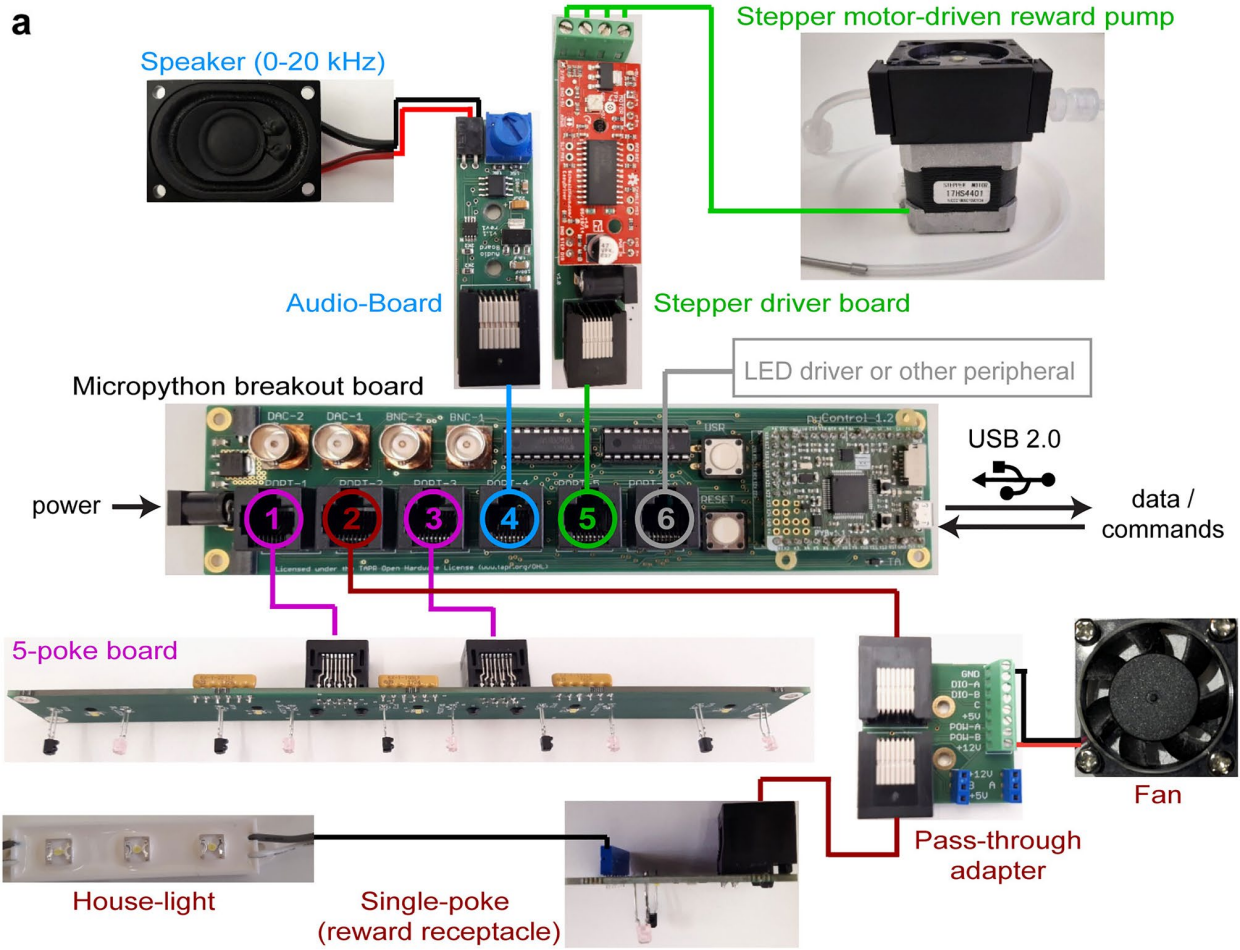
Connections between PCBs and peripherals. (**a**) Cabling scheme for the 5-choice operant box between PCBs and the peripherals they control showing named peripherals that plug into the indicated RJ45 (ethernet) ports (coloured numbers) of the pyControl breakout board. This includes the 5-poke board (RJ port 1 and 3), the single-poke receptacle board (port 2), the audio-board to deliver tones through a speaker (port 4), the stepper-motor control board to control a custom-made peristaltic pump for reward delivery (port 5), and an LED-driver for optogenetic stimulation through implanted LEDs (port 6, not shown); coloured lines represent RJ45 cables. The four BNC ports allow bidirectional communication with external devices through TTL pulses.

The pyOS-5 setup builds on *pyControl*, an open source system for controlling behavioural experiments, documented in a separate publication^10^ and at https://pycontrol.readthedocs.io. Briefly, pyControl provides users with flexible Python based syntax for defining tasks, and hardware for constructing operant setups. pyControl hardware comprises a breakout board and peripheral devices. The breakout board interfaces an Arm Cortex based Pyboard microcontroller with a set of ports and connectors. Peripheral devices connect to the breakout board via standard network cables (RJ45, 8-pin ethernet) and are used to construct behavioural setups. Peripherals either have a dedicated printed circuit board (PCB) to control them (pump, speaker, optogenetic LED) or constitute a PCB themselves (poke-hole arrays). For the 5-choice box, we developed a PCB integrating 5 infra-red break-beam sensors and 5 stimulus LEDs, used for the nose-poke ports on the 5 choice wall (part number py.026, Supplementary Table 1). Other pyControl devices used in the setup include a single nose-poke board used for the reward receptacle, a stepper motor controller board for the reward delivery pump, a special connector (pass-through adaptor) to breakout lines for other peripherals like a house-light, fan, or camera, or for TTL-signaling lines, and an audio board to play auditory stimuli (see Supplementary Table 1 for all PCBs used, Fig. 1a for their design and principal wiring). An LED driver board to control implanted LEDs for optogenetic modulation may also be added (Fig. 1)^11^. All pyControl hardware used for pyOS-5 is commercially available through the Open-Ephys store (http://www.open-ephys.org/store) at low cost (ca. 500 EUR per box), with design files available in the pyControl hardware repository (https://github.com/pyControl).

**Figure 2:**
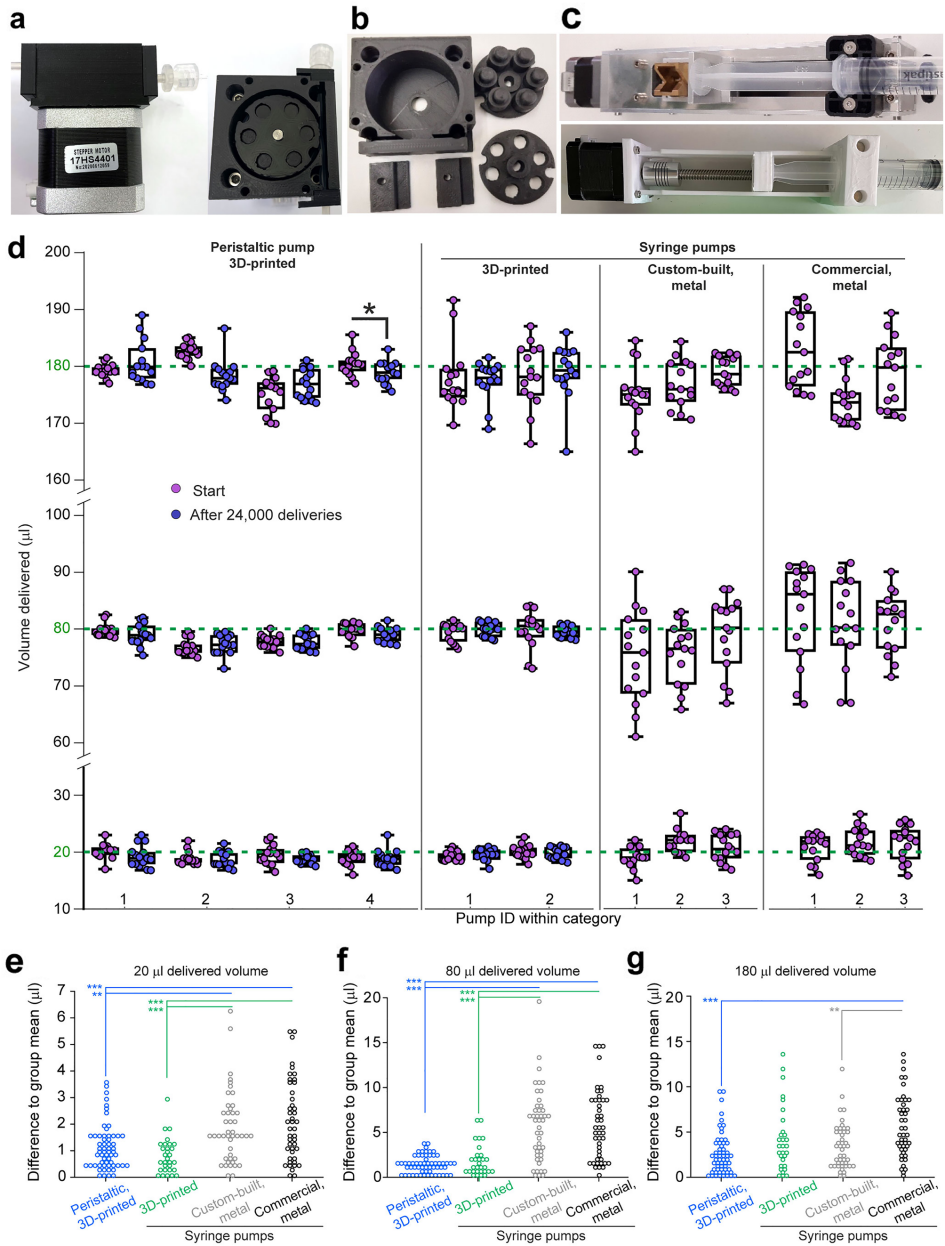
Peristaltic and syringe pumps for reward delivery. (**a**) Assembled peristaltic pump viewed from the side (left) and from top (right). (**b**) 3D-printed components that, together with a stepper-motor, are assembled to the peristaltic pump used for reward delivery and shown in **(a)**. (**c**) Custom-made syringe-pumps, made conventionally from metal parts (top) or majorly from 3D-printed parts (bottom), that can be used as alternative to the peristaltic pump and are driven by the same stepper motor. (**d**) Volumes delivered by individual peristaltic pumps (left), custom-made 3D-printed (mid left) or metal (mid right) syringe pumps, and commercially available syringe pumps (right, Med Associates, Inc.) when the output volume was set to approximately 20, 80, and 180 µl (green line) with 15 individual deliveries per volume and pump. Note that the setting of the output volume was identical for all individual pumps of a single kind, but differed between different kinds due to distinct coding of volumes by number of steps of the stepper motor or activated time for the commercial syringe pump (DC motor). For both types of 3D-printed pumps, stability of output was assessed by measuring volumes for 15 deliveries before (pink) and after (lilac) simulating 30 d of operation (24,000 deliveries, corresponding to 100 deliveries per session, 6 sessions per day); asterisk indicates comparison by paired unadjusted *t*-test (*P* = 0.041). (**e–g**) Variability of individually delivered volumes was assessed by converting values shown in **(d)** into the absolute value of the difference from the population mean of volumes measured for each type of pump (coded by colour). Asterisks indicate significant Tukey post-hoc comparisons after a significant effect of pump type in the overall one-way ANOVA calculated for each volume (*P* < 0.0001). ** *P* < 0.01, *** *P* < 0.001.

Additionally, IR capable CCTV-cameras were used to monitor animal behaviour through a CCTV system (Hikivision DVR HYB 5MP DS-7208HUHI-K1, 8 channel; or AVEESA HD-116 Pro H.264 DVR, 16 channel, iCCTV, Manchester, UK; Fig. 2f). Cameras were powered either from the pyControl breakout board or through separate power supplies.

### Open-source pumps for reward delivery

Use of liquid rewards requires accurate and reliable dispensing of small volumes of fluid. If commercially available peristaltic or syringe pumps were used, they would be a main cost driver during the acquisition of a custom-made operant box set-up. Solenoid valves can be used to deliver water rewards^10,11^, but are unsuitable for liquids with nutritional content as their remnants would lead to their rapid deterioration. Several solutions have been developed to deliver rewards in appetitively motivated rodent tasks, including pellet dispensers for sucrose pellets, dippers driven by solenoids (e.g. ENV-302R-S from Med Associates Inc., US), and syringe pumps (e.g. PHM-100A, Med Associates). Pellet dispensers and dippers have the advantage of imposing less effort for cleaning after testing but have the disadvantage of delivering only one size of reward, which is not useful for tasks like the 5-choice spatial working memory task (5CSWMT)^12^ or various probabilistic decision making tasks^11^ that use distinct reward sizes in different task phases. Pumps, by contrast, allow varying reward sizes but demand more cleaning at the end of the training day.

We developed pump designs that can all be driven by the same low-cost bipolar 2-phase 4-wire stepper motor (< 15 EUR; Supplementary Table 2), controlled by the same stepper motor driver board (py.022, Supplementary Table 1): two syringe pumps and a peristaltic pump (Fig. 2a–c; design files available at https://github.com/KaetzelLab/Operant-Box-Design-Files). The two syringe pumps differed in that one of them followed a classical design using solely metal parts (costing around 300 EUR including labour for machining), while the other incorporates 3D-printed parts for the complete frame except for the central metal jack and its coupling elements (lowering costs to ca. 65 EUR). The peristaltic pump solely deploys 3D-printed parts (Fig. 2a,b) screwed to the stepper motor, making it the most cost-effective (ca. 25 EUR including the motor).

We first investigated the precision of all three pumps when repetitively delivering approximately 20 µl of water, and compared this to a commercially available syringe pump used in operant box set-ups (PHM-100A, Med Associates). Dispensed water volumes were determined with a precision weighing balance (Quintix^®^ 224-1S, Sartorius Lab Instruments GmbH, G). The custom-made syringe pumps did not appear to exceed their commercial counter-part in terms of variance of the delivered volumes, and the peristaltic pump showed the least variability of volumes dispensed over 15 deliveries (range of coefficients of variation across all pumps of one type (CV): peristaltic pumps, 2.5–6.0%; 3D-printed syringe pumps, 3.3–4.2%; metal syringe pumps: 9.0–11.4%; commercial metal syringe pumps: 11.1–13.9%; Supplementary Fig. 1).

To scrutinize this preliminary conclusion, we repeated the experiment with three different delivery volumes (20, 80 and 180 µl) and re-tested the delivered volumes of the two low-cost (3D-printed) pumps after a simulated 30 d operation with 6 sessions per day (24′000 individual deliveries, one performed every 3 s). With the exception of one peristaltic pump where the two volumes differed at the 180 µl volume (but not at lower volumes), no statistically significant differences in delivered volumes were detected, suggesting a reasonable temporal stability of the set delivery volumes over time (Fig. 2d). To quantify the qualitatively observed differences in the variability of individual delivery volumes between pumps (Supplementary Fig. 1, Fig. 2d), we converted every volume into the absolute value of its difference to the group mean across all pumps of each type. At all three delivery volumes, the average difference to the group mean observed at individual deliveries was lower with the peristaltic pump than with at least one of the metal-based syringe pumps; and likewise, the 3D-printed syringe pump was more precise than its metal counterparts at the low and medium volume (Fig. 2e–g). This performance of custom-made pumps opens the possibility for cost-effective reward delivery solutions that can be easily integrated into any operant system using low-cost microcontrollers.

### Design of the 5-choice operant box

**Figure 3:**
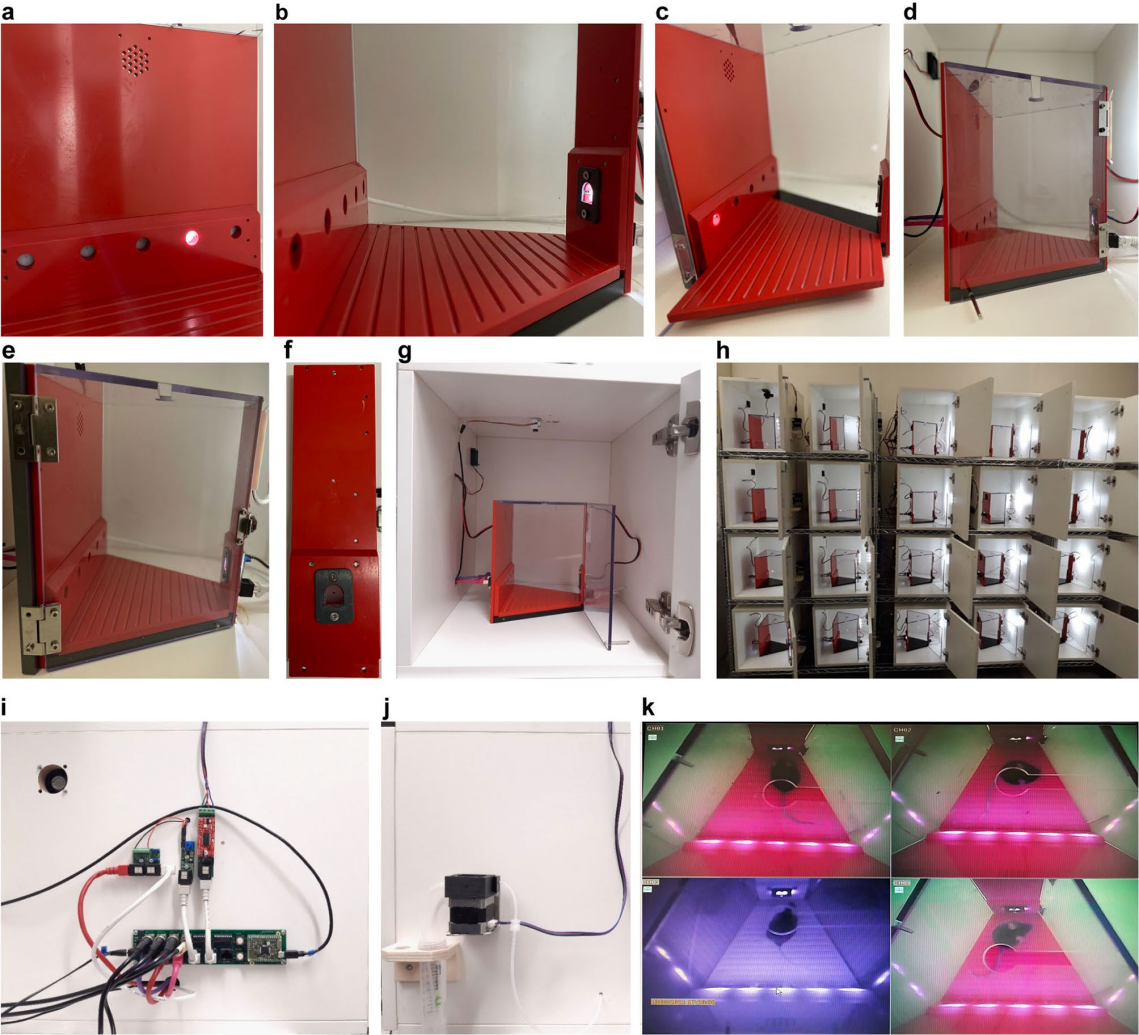
Design of inner and outer box. (**a**) 5-choice wall with one poke-hole illuminated. Note perforation at top for speaker. (**b**) 5-choice (wall) and receptacle (right) wall, showing protruding design of poke panel with angled finish at top and elevated position of poke holes; receptacle poke illuminated. (**c**) Easily washable, sliding floor with groves for fast turn-over of subjects between runs. (**d,e**) Side view of complete operant box with two different door designs: door opening on the left and fixed in place by a magnet at its bottom that also constitutes the door handle **(d)**, or door opening on the right and fixed by snap-lock **(e)**. (**f**) Frontal view on isolated receptacle wall with grey frontal spacer layer (2 mm thickness) that shields break-beam. (**g**) Operant box within outer sound-attenuating cubicle, including all cabling required for operation. (**h**) Set-up of 20 stacked operant boxes within their cubicles, house-lights illuminated. (**i,j**) Left (**i**) and right (**j**) outer side walls of the cubicle with mounted PCBs on the left (compare cabling to Fig. 1a) and peristaltic pump and reward container on the right. (**k**) Visual monitoring of animal behaviour through low-cost CCTV cameras and a 16-channel CCTV system (only 4 boxes shown). Boxes that are currently not illuminated due to time-outs are video-recorded via infrared and hence appear in grey-purple.

A 5-choice design (Fig. 3a) was chosen to take advantage of the many behavioural paradigms that have been implemented with this layout, including assays for attention, perseveration and motor impulsivity, using the classical 5-choice serial reaction time task^13^ or the related continuous performance test^14,15^, but also decision impulsivity^16–18^, rule-shift learning^19^, working memory^12^, and motivation^2^. The operant box can also be modified by replacing the 5-choice wall (plastic components and 5-poke PCB) with a different layout such as a 2 + 2 design^11^. The 5-choice wall also mounts a speaker for auditory stimulation (Fig. 3a; see Supplementary Video 1 for operant box assembly process).

We implemented a set of design considerations detailed in Table 1 (see also Fig. 3a–j). Design files for all plastic components of the operant box can be downloaded from https://github.com/KaetzelLab/Operant-Box-Design-Files. They were manufactured by CNC machining, laser cutting, or 3D printing, as indicated in the design files. The main Perspex and PVC components were fabricated in-house using CNC-machining and drilling. Perspex acrylic panels (5 mm white, Opal030 and Opal050) that served as light attenuators and spacers placed between the 5- and single-poke PCBs and the inner wall of the operant box were obtained from CutLaserCut (Camberwell, UK). A further spacer for the 5-poke PCB (preventing direct contact between LED and Opal-layer) were generated by 3D-printing. See Supplementary Video 1 and the Supplementary Construction Guide for illustrated step-by-step instructions.

**Table 1:** Design considerations for inner and outer box.

Design concern	Design solution
Minimize area of exploration, enhance focus on 5-choice wall	Trapezoidal foot-print (Fig. 3b–e)
Minimize distracting cues	Homogeneous interior colour and surfaces, speaker and house-light mounted outside of the inner box, most cabling and electronics hidden behind opaque walls; no internal protrusions to discourage climbing (Fig. 3a–d)
Homogeneous, diffuse, and reproducible ambient illumination	House-light (white LED module) fixed centrally at the back-side of receptacle-wall, facing away from the operant box, producing ca. 20 lx diffuse illumination inside the box (Fig. 3d,e,h)
Maximise ease of movement in box	Machined plastic floor with shallow 45° groves to collect urine (instead of a metal grid)—no trip hazard (Fig. 3b,c)
Testing capability for a wide range of behavioural functions	5-choice design of poke-wall allows testing of attention, motivation, perseveration, working memory, impulsivity, and cognitive flexibility; the whole 5-choice wall can also be replaced by a wall with a different poke-layout at relatively small cost
Naturalistic rewarding possible (unifying the event of making a choice and collecting the reward it implies)	Every poke-hole of the 5-poke wall has the same through-hole design as the reward-receptacle so that reward can be delivered directly at the choice hole; multiple low-cost pumps per box (a total of three from the break-out board, more if necessary though a port expander) can be controlled independently for port-specific reward delivery
Suitability for tethered animals	Extended height of inner (20 cm) and outer (38 cm) box, slit & hole in roof-top of inner box; total height of outer box still allows vertical stacking of 4 boxes for efficient use of lab space (Fig. 3d,e,g,h)
Suitability for implanted animals	Shallow poke-hole recesses (7–8 mm), shallow position of break-beam (2–3 mm from surface), protruding poke-hole panel (Figs. 3a,f, 6a,d); depth of break-beams is adjustable by choice of spacers
Suitability for animals with headstages or miniscopes	As above; additionally: slightly elevated poke-holes and reward-receptacle (30 mm above ground; Fig. 3a,b) ensure head is tilted backward during poke-entry so implant does not get in the way. Poke-hole panels terminate just above hole to allow CMOS-chip from miniscopes to protrude above it (Fig. 3a,b); upper edge of protruding poke-walls angled at 45° to prevent climbing (Fig. 3a,b,f)
Fast cleaning and turn-over between animals	Removable (sliding) floor—dirty floor replaced by clean one within few seconds (Fig. 3b,c); inner door closes with magnets or snap lock (2 designs available; Fig. 3d,e); single outer enclosure with door on soft-close hinges (Fig. 3g, right)
Sound-attenuation	Melamine-coated MDF walls for outer box (Fig. 3g,h); sufficient space to fit 30–50 mm diameter sound-absorbing material at inside, if needed
High discriminability of cues for fast learning	Relatively wide distance between 5-choice holes (4 cm; Fig. 3a)
Compact design of box & electronics; easy installation	All PCBs, electronics and reward containers are mounted on the outside opaque walls of the inner box or an external wall of the outer box (Fig. 3g–j); only 3 cables connect each box externally: power adapter, USB-data cable, camera

### Design of the outer box

The outer enclosure enables tight control over visual and auditory stimulation and background (Fig. 3g,h). The outside walls also mount PCBs and cabling to control the peripherals, the peristaltic pump and reward container (Fig. 3i,j), while the inner ceiling holds a CCTV camera to monitor behaviour online (Fig. 3k). 19 mm sealed MDF was used as wall material to provide basic sound insulation, but the size of the box also permits sound-absorbing material of 30–50 mm thickness to be applied to all six inner walls if needed. The box size was chosen to allow efficient use of lab space (e.g. permitting the stacking of 4 boxes above one another in racks; Fig. 3h), while also being high enough to provide flexibility for optical fibres or cables tethered to animals (Fig. 3d,e,g). Design drawings are available from https://github.com/KaetzelLab/Operant-Box-Design-Files.

### Design of the graphical user interface

**Figure 4:**
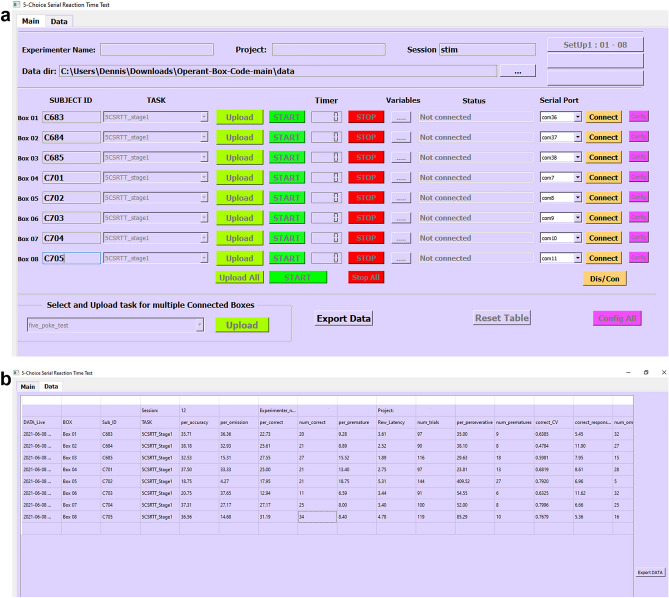
Graphical User Interface (GUI). (**a**) Main panel of the multi-control pyOS-5 GUI that allows to connect and control up to 8 boxes at the same time, whereby each box can run a different task file, if necessary. Multiple GUIs can be run in parallel. (**b**) Data panel of the same GUI displaying relevant behavioural performance parameters that are continuously updated and that can be directly exported as csv-file at the end of the experiment.

We developed a graphical user-interface (GUI, Fig. 4a,b; freely available from https://github.com/KaetzelLab/Operant-Box-Code) specifically for the pyOS-5 boxes. This GUI was created using Qt Designer (https://www.qt.io/) and inherits most of its key functions from the pyControl framework and its own generic GUI^10^. While the generic pyControl GUI^10^ is designed to provide maximum flexibility for controlling arbitrary behavioural assays, the pyOS-5 GUI is specialised for use with specific 5-choice based tasks, and provides dedicated functionality to extract task-specific performance measures for online monitoring and subsequent offline analysis. The behavioural hardware is compatible with both GUI’s, and both use the same Python-based task definition files, facilitating reproducibility and communication of task logic^10^. Both GUIs allow different subjects to be run on different task configurations in parallel, but the primary mechanism for this in the pyOS-5 GUI is specifying different task files for each subject, whereas the pyControl GUI primarily uses task variables for this.

We provide ready-to-use scripts for a wide range of behavioural tasks including all common training stages and challenge protocols of the 5-CSRTT^13,19^, the continuous performance test^15^, the 5-choice delayed-matching-to-position operant spatial working memory (5-CSWM) task^12^ and a 2-choice delayed-non-matching-to-position version of it, and a rule-shift task^19^ (available from https://github.com/KaetzelLab/Operant-Box-Code). One GUI window can control up to 8 boxes, and as many GUI windows as necessary can be opened and run in parallel in order to control large set-ups (we use a 24-box set-up, controlled from three GUI windows). In the pyOS-5 GUI all relevant task performance parameters are continuously updated to monitor animal behaviour online (Fig. 4b). At the end of a session, the final values of all parameters relevant for a given task paradigm can be directly and quickly exported as csv-file. In addition, a further results csv-file is generated that records every single event (e.g. nose-poke into a specific hole, delivered stimulus or reward) with its time-stamp to enable easy analysis of detailed trial-by-trial behaviour. This also allows the offline correlation of individual behavioural events with activity traces gained from simultaneous physiological measurements (e.g. miniscope imaging or electrophysiological recordings) during later analysis in a separate software (see below).

## Animals and behavioural testing

### Animals

All experiments were approved by the Federal Ethical Review Committee (Regierungsprädsidium Tübingen) of Baden-Württemberg (licence numbers TV1344 and 1399), performed in accordance to the German Animal Rights Law (Tierschutzgesetz) 2013 and are reported in accordance with the ARRIVE guidelines. Animals were group-housed (3–5 mice per cage) in Type II-Long individually ventilated cages (Greenline, Tecniplast, G), enriched with sawdust, sizzle-nest™, and cardboard houses (Datesand, UK), and subjected to a 13 h light/11 h dark cycle. Mice were kept under food-restriction at 85–95% of their average free-feeding weight which was measured over 3 d immediately prior to the start of food-restriction at the start of the behavioural training. Water was available ad libitum. In this manuscript, we present data from five cohorts that were subsequently used for other studies. Four cohorts were trained on the 5-CSRTT, and one on the 5-CSWM task (see below and Supplementary Fig. 2 for time lines of experiments). The first cohort of 18 male C57BL/6 J mice, aged 12–13 weeks at the beginning of training was used for initial testing and validation of the system (cohort 1). Secondly, two cohorts of 20 (cohort 2) and 37 (cohort 3), respectively, male B6.FVB(Cg)-Tg(Rbp4-cre)KL100Gsat/Mmucd (Rbp4-Cre) mice^20^ (maintained on a C57BL/6J background) were used to compare task acquisition on the pyOS-5 system with a commercial system. These were trained by the same experimenter and according to the same 5-CSRTT training schedule, starting at the age of 2–7 months, in either the pyOS-5 system (cohort 2) or an equivalent commercial nose-poke-based system (ENV-307A-CT with ENV-115C-A 5-choice wall; Med Associates, Inc., US; cohort 3). The remaining cohorts were used to assess compatibility of the system with optogenetic manipulations and physiological recordings. Four male C57BL/6J mice (cohort 4) were pre-trained in the 5-CSRTT, then transfected into the dorsal hippocampus (dHC) with an AAV8-vector containing either a CamKIIα-Jaws-GFP or a CamKIIα-ArchT-GFP construct (2 mice each; UNC vector core), implanted with an 200 µm diameter fibre-optic cannula (ThorLabs, UK) into the right dHC, and with two single polyimide-insulated tungsten wires (50 µm diameter, Wiretronic, USA) into the right prefrontal cortex (PFC, AP + 1.7–105 1.8 mm, ML + 0.25–0.35, 1.7–1.9 mm below pia) and dHC (AP −2 mm, ML + 1.5 mm, −1.3 mm below pia); a reference wire (120 μm) was implanted above the left frontal cortex, and a ground screw above the left cerebellum; implants were secured using self-adhesive resin cement (Breeze™ Pentron clinical, US). After two weeks of recovery on free food, mice commenced with 5-CSRTT training and were used for testing with a fibre-optic tether (200 µm core, ferrule-sleeve connector, Thorlabs, GB) and optogenetic modulation using continuous illumination at ~ 3.5 mW with either a 532 nm DPSS laser (for ArchT) or a 635 nm diode-laser (for Jaws; both CNI Lasers, CN). A separate cohort of 11 wild-type mice (cohort 5) was trained in the 5-CSWM task^21^, implanted with polyimide-insulated tungsten wires in the PFC and dHC (as described above) as well as into the ventral hippocampus (vHC; AP -3.1–3.2, ML 2.9–3.0, 3.4 mm or 3.8–3.9 mm below pia) and mediodorsal thalamus (MD; AP -1.2, ML 0.3, 2.7 below pia), in addition to frontal and cerebellar skull screws for reference and ground, respectively. After 2–4 weeks of recovery, training commenced. For the present paper the last four days of training *without* mounted headstage (Intan, US) and the subsequent first five days *with* mounted headstage are analysed, while further data from these animals, alongside more detailed methodical description of the surgery, behavioural training, and electrophysiological data acquisition are described elsewhere^21,22^.

### 5-choice-serial-reaction-time task (5-CSRTT)

The 5-CSRTT training protocol was identical to what we previously described (illustrated in Supplementary Fig. 2)^23^. In brief, after initiation of food-restriction, mice were accustomed to the reward (strawberry milk, Müllermilch™, G) in their home cage and in the operant box (2–3 exposures each). Then, mice were trained in a simplified operant cycle in which all holes of the 5-poke wall were illuminated for an unlimited time and the mouse could poke into any one of them to earn a 40 µl milk reward subsequently disposed from the illuminated receptacle (habituation training). If mice attained at least 30 rewards each in two consecutive sessions, they were moved to the 5-CSRTT. During this training, mice transitioned through five stages of increasing difficulty, based on reaching certain performance criteria in each stage (Supplementary Table 3). The difficulty of each stage was determined by the length of time the stimulus was presented (stimulus duration, SD) and the length of waiting time between the end of the previous trial and the stimulus presentation of the next trial (inter-trial-interval, ITI).

In the principle 5-CSRTT operant cycle, the ITI was initiated by the removal of the snout of the animal from the reward receptacle after collection of the reward or by the end of a time-out period (see below). The ITI was followed by the illumination of one hole of the 5-choice wall for the SD determined by the task stage. At the end of the SD, the light was turned off and the animal was allowed another 2 s (limited hold, LH) to poke into the previously indicated hole. If this hole was poked during the SD or LH period, this was counted as a *correct response* and rewarded by immediate delivery of 20 µl milk at the receptacle hole which was accompanied by its illumination, and followed by the initiation of a new ITI. If the animals either poked into any 5-choice hole during the ITI (*premature response*), poked into a non-illuminated hole (*incorrect response*) during the SD or limited-hold time, or failed to poke throughout the trial (*omission*), trials were not rewarded but instead terminated immediately with a 5 s time-out period during which the house light was turned off. The relative numbers of such response types were used as performance indicators measuring premature responding [*%premature* = 100 × (number of premature responses)/(number of trials)], sustained attention [*accuracy* = 100 × (number of correct responses)/(number of correct and incorrect responses combined)], and task participation [*%omissions* = 100 × (number of omissions)/(number of trials)]. In all stages and tests, sessions lasted 30 min and were performed once daily at the same time of day.

### 5-choice-spatial working memory task (5-CSWMT)

The training of cohort 5 in the 5-CSWMT is described in detail elsewhere^21^, as is comparable data from the same task acquired in commercial boxes^12,24^. Briefly, each trial of this delayed-matching-to-position (DMTP) spatial working memory assay consists of a *sample phase* (SP) during which the mice have to detect and poke an illuminated hole (as in the 5-CSRTT), a *delay phase* during which mice obtain a small reward from the receptacle (10 µl strawberry milk), and the *choice phase* (CP) during which mice are presented with two illuminated holes at the 5-choice wall (see illustration of task cycle in Supplementary Fig. 2). One of these two indicated holes is the one that was also illuminated in the prior SP and has to be poked again in the CP in order to obtain a large reward (60 µl). Incorrect responses or omissions in the SP or CP are followed by abortion of the trial in form of a 5 s time-out and a subsequent ITI. In contrast to the 5-CSRTT, the task is run with the house-light *off* in the default mode, and *on* during time-out periods. Animals were trained through multiple, increasingly more difficult stages (with parameters specified in Supplementary Table 4) and data presented here was obtained on the baseline stage 5, which has a maximum SD of 10 s in the SP and of 5 s in the CP, and a delay after the collection of the SP-reward of 2 s before the CP starts.

## Results

We here present data illustrating operant testing of untethered and tethered mice in a pyOS-5 set-up. Note that we already published 5-CSRTT data from a small pilot-cohort of eight mice that were trained up and tested in a prototype of the pyOS-5 boxes—that data demonstrated the relative selectivity of parametric test protocols that challenged either attention (reduction of SD, sound distraction) or impulsivity (fixed or variable increase of the ITI) and the responsiveness of these behaviours to atomoxetine^10^.

### Behavioural validation in the 5-CSRTT

**Figure 5:**
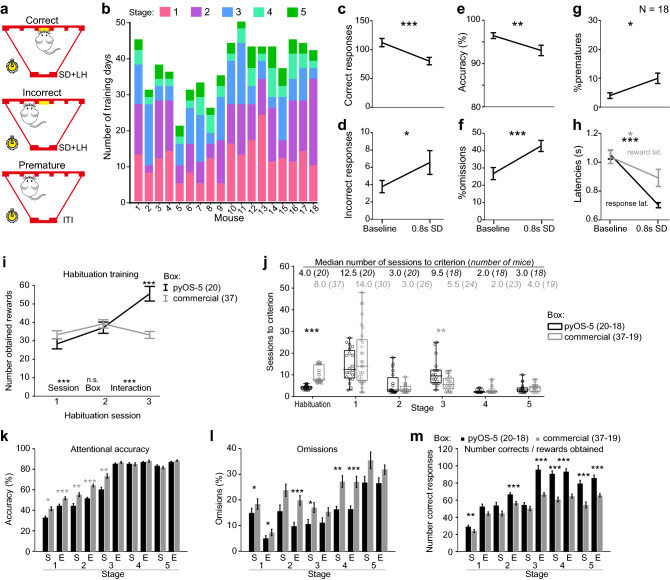
5-CSRTT training and performance. (**a**) Three principal response types of the 5-CSRTT, as indicated. Schemes were drawn in CorelDraw Graphics Suite 2021 (https://www.coreldraw.com). (**b**) Number of training sessions needed by each of the 18 mice in the cohort to transition through the 5 training stages (colour-coded) from the first training day with 65 lx poke-hole illumination (day 14 on stage 1; see Supplementary Fig. 3) until reaching of criterion on stage 5. (**c–h**) 5-CSRTT performance during the final training stage (5, baseline) and an attention challenge (0.8 s SD); shown are indicators of sustained attention and task participation, namely total number of correct **(c)** and incorrect **(d)** responses **(c)**, their ratio (accuracy, **e**), relative omission rates **(f)**; an indicator of motor impulsivity (relative number of premature responses, **g**); and reward (black) and response (grey) latencies **(h)**. Lines display mean ± s.e.m. Asterisks in **(c–h)** indicate significant within-subject comparisons relative to the baseline (2 s SD, 5 s ITI) with paired-samples *t*-tests. (**i**) Number of rewards obtained in the first 3 sessions of habituation training; assessment at bottom reflects RM-ANOVA. (**j**) Number of sessions needed to reach criterion on each training stage shown for individual animals and as box-plots for the whole group, median number for each group (black, pyOS-5; grey, commercial system) stated on top with contributing *N*-numbers in brackets; *N* vary because not all animals were trained continuously to stage 5. (**k–m**) Key performance parameters that determine stage transitions, named above each panel, are displayed as mean ± s.e.m. across animals. Each contributing data point was calculated as average of the first two (S) or last two (E) days an animal was trained on the indicated stage. Asterisks above data in **(i**), (**j)**, (**k–m**) indicate Sidak post-hoc, MWU, and Bonferroni-adjusted *t*-tests, respectively. Asterisks in black indicate that mice trained in pyOS-5 outperform mice trained in the commercial box. *P < 0.05, **P < 0.01, ***P < 0.001.

In this study, we first trained a larger cohort of 18 male C57BL/6J mice in the 5-CSRTT (see “Methods” for training schedule, and Fig. 5a and Supplementary Video 2 for an illustration of the task). As light levels and colour of 5-choice wall stimuli vary greatly between commercial systems (we have previously used defaults of 215 lx white light in one system (Campden Instruments Ltd.^25^) and 4 lx of yellow light in another (Med Associates Inc.^19,23,24^), we started with a high illumination of 280 lx of white light as measured in front of the poke-holes. We observed qualitatively that some animals would refrain from poking into the hole even when sitting right in front of them and that no training progress was evident over 4 days as assessed by repeated-measures (RM) ANOVA (Supplementary Fig. 3). Taking advantage of the exchangeability of the 5 mm Opal-spacer layer that is available in different transmissivity (CutLaserCut, GB) which can be further adjusted with tape, we reduced light output to 95 lx of either yellow or white light, and, on session 14, further to 65 lx white light, which was kept because of the observed performance increase (Supplementary Fig. 3; all animals trained on stage 1 throughout), and was used for all subsequent training and experiments in this and the other cohorts. Mice of this cohort required on average 26.5 days (range: 14–37 days) from the end of stage 1 until reaching criteria on the final stage 5 (baseline; Fig. 5b).

Once animals had achieved criterion on the baseline stage indicating stable performance, we conducted a widely used challenge in the task^19,23,25–27^ which demands high levels of sustained attention: shortening of the SD from 2 to 0.8 s. As predicted, this protocol led to a decrease of *correct* and an increase of *incorrect* responses, thereby causing a decrease in attentional accuracy (Fig. 5c–e). As we observed before^10,19^, also omissions and, mildly, premature responding increased in this paradigm, while response latency decreased (Fig. 5f–h). The absolute levels of baseline behaviour also illustrate that the pyOS-5 system allows animals to achieve high numbers of correct responses (112.2 ± 7.1, mean ± s.e.m.) and consequently total trials (161.9 ± 2.6) in 30 min, while omission rates remained relatively low for murine standards (26.4 ± 3.5% at baseline, Fig. 5c,f).

In order to quantitatively compare the achieved training performance between our and a commercial nose-poke-based system (Med Associates, Inc.) on the final 5-stage training protocol (without the initial changes in light levels done in cohort 1), we analysed training data of two cohorts (2 and 3) that were of the same genotype (Rbp4-Cre, phenotypically wildtype) and trained by the same experimenter according to the same 5-stage schedule (Supplementary Table 3). These cohorts were trained for a separate study which required surgery post training, but all mice had at least 42 training sessions without breaks of more than 2 d. Surgery was performed cage-wise, such that some animals were operated before completing all 5 training stages and some mice were excluded due to extremely slow progress in stage 1 (in commercial boxes only). This resulted in two mice from cohort 2 (trained in the pyOS-5 system, *N* = 20) and 18 mice from cohort 3 (trained in the commercial system, *N* = 37) contributing data to only a subset of training stages (2.6 ± 1.5, mean ± S.D., including habituation). As these mice were typically ones with slower training progress, the analysis of later training stages may be somewhat biased in favour of the commercial system where more mice were not trained until stage 5 continuously. Mice trained in the pyOS-5 system showed a higher number of achieved responses and hence rewards by day 3 of the habituation training and therefore moved to stage 1 of the 5-CSRTT significantly earlier, compared with mice trained in the commercial system (Fig. 5i,j). Conversely, they needed significantly longer to reach criterion on stage 3, while not differing significantly in the required time on the other four training stages (Fig. 5j). The sum of median training days across stages was 34 in pyOS-5 boxes and 37 in commercial boxes (not taking into account 7 mice trained in commercial boxes that did complete stage 1 within ≥ 26 [range: 26–55] sessions on that stage and all stages within ≥ 42 [range: 42–65] sessions in total; Fig. 5j).

Given that the three key variables accuracy, %omissions, and the absolute number of correct responses determine the transition between stages (Supplementary Table 3), we next investigated performance differences between cohorts 2 and 3 in those parameters measured at the beginning and end of each stage (average of two sessions each). Compared to animals trained in commercial boxes, mice trained in pyOS-5 boxes displayed significantly lower accuracy in the early 3 stages, but showed a lower omission rate throughout stages and a higher number of correct responses (i.e., obtained rewards) in the last 3 stages (Fig. 5k–m). This suggests, that it is mainly the slower improvement in accuracy over the training sessions that causes the slightly larger number of required training days in the pyOS-5 boxes on stage 3, while this system generally benefits a higher task participation. Hence, given that mice reach equal accuracy in stages 4 and 5 in pyOS-5 boxes, training may be faster, if the accuracy criterion in stage 3 is somewhat relaxed, in future studies.

### Behavioural validation in the 5-CSRTT using tethered animals

**Figure 6:**
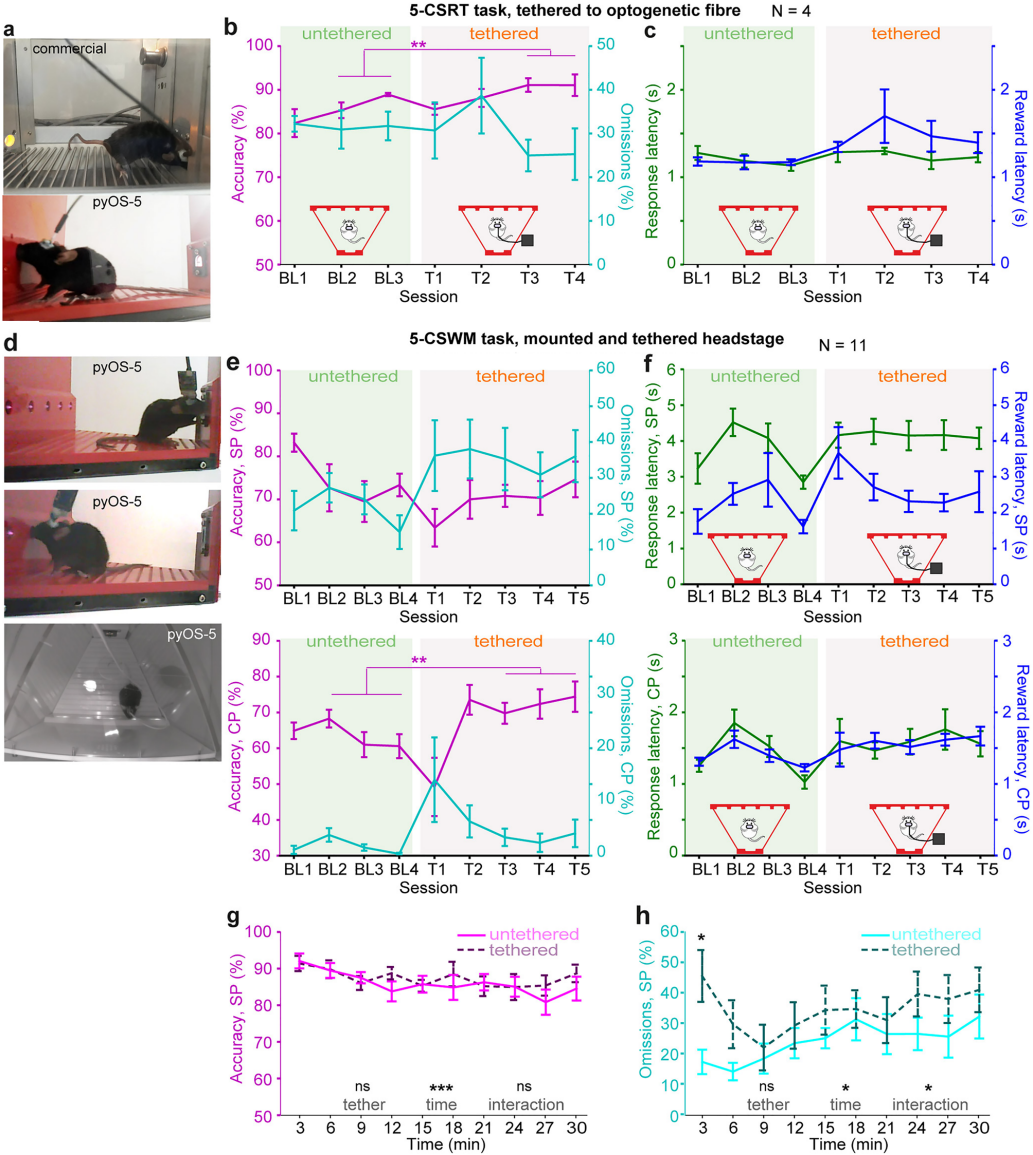
5-CSRTT performance under tethered condition. (**a**) Mouse implanted with a fibre-optic cannula and tethered to a fibre patch cord collecting reward from the receptacle of a commercial operant box (top) or poking into 5-choice wall in a pyOS-5 box (bottom). (**b,c**) 5-CSRTT performance parameters in untethered (BL1–3) and tethered (T1–T4) training in consecutive sessions indicating similar or higher levels of attentional accuracy (**b**, purple), task participation measured by omission rates (**b**, cyan), mean duration to respond to the correct illumination, response latency (**c**, green), and duration to reach the reward receptacle after a correct response, reward latency (**c**, blue), if comparing the average of the last 2 days under each condition (paired *t-*test). (**d**) Mouse implanted and tethered via a headstage and SPI-cable collecting reward (top), poking into a 5-choice hole (middle) and waiting in front of the 5-choice wall (bottom) of a pyOS-5 box. (**e,f**) 5-CSWM task performance parameters from the sample phase (SP, top) and choice phase (CP, bottom), as stated on colour-coded y-axes, during consecutive training sessions conducted untethered (BL1–4) or tethered (T1–T5) showing similar or higher performance in the tethered state when comparing the averages of the last 3 days under each condition (paired *t*-test). (**g,h**) Averaged SP-data from the last three 5-CSWMT sessions conducted under each condition as shown in (**e,f**), BL2–4 and T3–5, calculated in 3-min intervals across the session to investigate signs of fatigue. Effects stated at the bottom of each panel refer to RM-ANOVA, and asterisk above data lines shows Sidak post-hoc comparison after significant tether-time interaction. Data shown as mean ± s.e.m. throughout. *P < 0.05, **P < 0.01, ***P < 0.001.

Next, we qualitatively evaluated the possibility that animals that are implanted and tethered to an optical fibre can easily access the poke holes and reward receptacle (Fig. 6a, also showing the contrast to a commercial system; Supplementary Video 3). For additional validation of the suitability of the operant boxes for tethered mice, we trained four C57BL/6 mice in the 5-CSRTT, implanted them with a fibre-optic cannula, and trained them further after a period of recovery (cohort 4). Once the mice had reached stage 5 (baseline; Supplementary Table 3), they were trained for three further days *untethered*, and subsequently for a further four days *tethered* unilaterally to an optical fibre. We compared key performance variables like attentional accuracy and omissions as well as response and reward latencies (indicating attainable speed of movement in this case) in sessions 3 and 4 *with tether* (i.e. after a 2-day acclimation period with the tether) to the average performance during the last two days *without a tether.* Mice did not perform worse in the tethered mode compared to the untethered mode according to any of the variables, and even performed better in terms of accuracy (*P* < 0.05, paired *t*-test; Fig. 6b,c).

To further investigate the suitability of the pyOS-5 boxes for tethered animals, we conducted a similar analysis for a group of 11 wildtype mice that had been trained in the 5-CSWM task, implanted with depth electrodes, and were trained until the baseline stage (5, Supplementary Table 4) of the task. After at least four training sessions on this stage, these mice were trained for a further 5 d on this stage with a mounted headstage (Intan, 24 × 15.5 mm, ca. 1.3 g including custom-made connector) tethered with an SPI cable (Intan; Fig. 6d). Again we compared attention- and working memory-related performance variables and latencies after a 2-session acclimation period, comparing the average of sessions 3–5 *with tether* to the average of the last 3 session *without a tether* (Fig. 6e,f). Tethered mice performed as well as untethered mice and even showed higher working memory accuracy (Fig. 6e,f).

We scrutinized this conclusion further by analysing attentional accuracy and omissions, both measured in the sample phase (SP) over the time of the session (3 min time intervals, again using 3-session averages as before)—taking advantage of the fact that pyControl records every single behavioural event to allow such fine-grained analysis. While there was again no significant effect of *tether*, there was a significant effect of *time* on both variables and a *tether-time interaction* for SP omissions (*P* < 0.05; RM-ANOVA), mainly driven by higher omissions in the first interval in tethered animals (*P* = 0.01, Sidak-adjusted post-hoc test for simple main effects; Fig. 6g,h). This pattern suggests that the animals can move and respond unhindered enough in the boxes (Supplementary Video 3) so that even with such a large headstage, tethering does not speed up normal fatigue occurring across the session, but only demands an acclimation to the tether at the beginning of the session.

### Integration of behavioural experimentation with electrophysiological recordings

**Figure 7:**
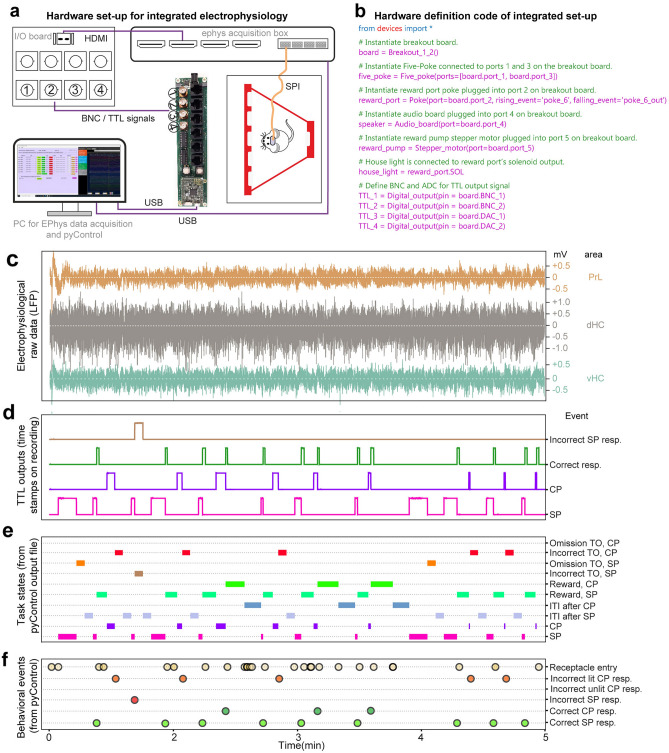
Integration of behavioural testing with electrophysiology. (**a**) Wiring scheme integrating the pyControl system with an Open-EPhys acquisition system. (**b**) Hardware definition code determining all peripheral circuit boards and the four TTL-outputs (additional comments in green). (**c–f**) Temporally aligned electrophysiological LFP data from the indicated and colour-coded brain areas **(c)**, TTL-outputs encoding behavioural events sent from pyControl and recorded as time-stamps by the electrophysiological acquisition software **(d)**, and task states **(e)**, and behavioural events **(f)** recorded by pyControl. *TO* time-out, *resp* response.

In cohort 5, we further established and evaluated the suitability of the pyOS-5 system for integrated electrophysiological recordings during operant box tasks. This requires precise temporal alignment of electrophysiological recordings with set task states and behavioural events. To physically interface pyControl hardware with physiological recording systems, the breakout board has four standard BNC-connectors (Fig. 7a) which can be defined as digital outputs or inputs in the hardware definition-file (Fig. 7b). Used as outputs, these allow TTL-signals to be triggered from the task script, e.g. at the onset of a given state or behavioural event, and recorded as time-stamps by the electrophysiology acquisition system (Fig. 7a). (Note that more than 4 output lines can be generated by using break-out adapters, py.024, Supplementary Table 1.) As pyControl also records millisecond timestamps for all behavioural events, these can be aligned to physiology traces during offline analysis using the TTL pulses recorded alongside physiology data as a reference point. Figure 7 illustrates the alignment of local field potential recordings from three different brain regions (Fig. 7c) with TTL-based time-stamps recorded by the electrophysiological acquisition software (Open-EPhys; Fig. 7b), behavioural states determined by the task script (from the pyControl record; Fig. 7e) and the animal’s active responses (from the pyControl record; Fig. 7f) during 5 min of a 5-CSWMT session. Also note that no artefacts are evident in the electrophysiological recording as the animal enters choice-pokes or the reward receptacle (Fig. 7c,f; Supplementary Fig. 4). The high stability and quality of such recorded data is also demonstrated by its suitability to decode individual working memory-based choices on a trial-by-trial basis in the same cohort^21^.

### Integration of behavioural experimentation with optogenetic stimulation

**Figure 8:**
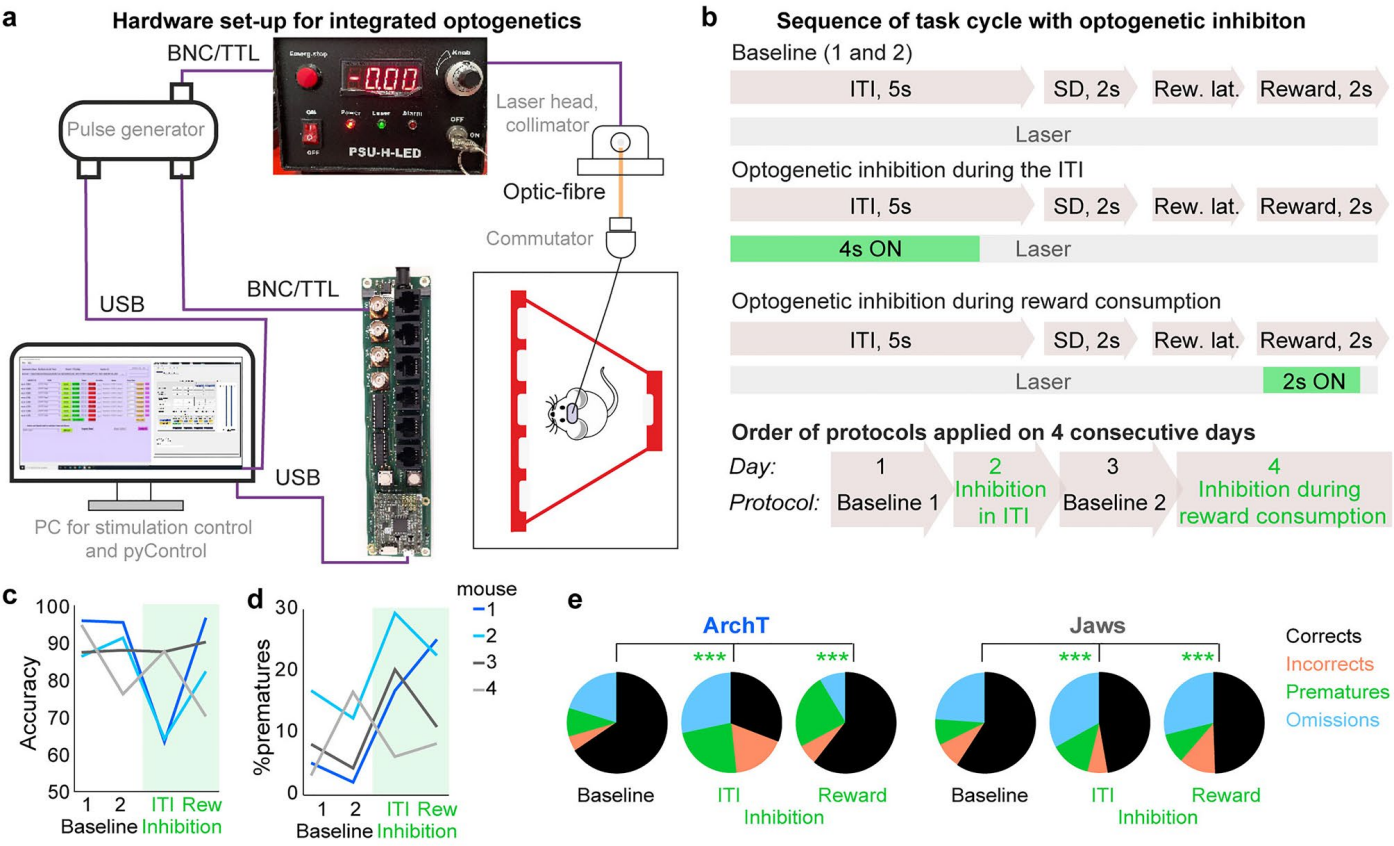
Integration of behavioural testing with optogenetics. (**a**) Wiring scheme for controlling a pulse generator and connected laser from pyControl through a BNC/TTL-output from the breakout board. (The hardware definition file for this arrangement is identical to that shown in Fig. 7b.) (**b**) 5-CSRTT task-cycles (illustrated for the case of a correct response) with either *no* optical stimulation (baseline sessions 1 and 2), unilateral optical modulation of the dHC during the first 4 s of the ITI or during the first 2 s of reward collection (from top to bottom). Below, the order in which the four protocols were conducted is shown. (**c,d**) Attentional accuracy **(c)** and premature responding **(d)** of each mouse (colour-code in **d**); protocols with optical modulation indicated in green. Note that mice 1 and 2 were transduced with ArchT, while the remainder were transfected with Jaws. (**e**) Share of each of the four response options (colour code on the right) in the indicated condition (baseline sessions 1 and 2 merged); asterisks indicate chi-square test between baseline and each of the stimulation conditions. ***P < 0.001.

In cohort 4, we further evaluated the suitability of the pyOS-5 system for integrated optogenetic modulation during operant box tasks. To this end, TTL-outputs can be generated directly from within the pyControl task file to either switch a laser power supply directly (for continuous or simple pulsed stimulation) or trigger a pulse generator (for temporally more complex stimulation), at a specific point during a task cycle (Fig. 8a,b). In our pilot cohort of two ArchT- and two Jaws-transduced mice (for unilateral optogenetic inhibition of the dorsal hippocampus), we applied a sequence of 4 sessions on consecutive days, whereby no optical modulation was conducted in sessions 1 and 3 (termed baseline; tether only), optical modulation during the first 4 s of the ITI was conducted in session 2, and optical modulation during the first 2 s of the reward collection preceding the ITI in session 4. Qualitatively, ArchT-transduced mice showed a notable decrease of attentional accuracy and increase of premature responses especially in the session where modulation was applied during the ITI (Fig. 8c,d). The pattern of frequencies of each of the four response options (corrects, incorrects, prematures, omissions) differed significantly from baseline in each of the optical modulation conditions (Fig. 8e). Capitalizing on the fact that pyControl records every single behavioural event, we also analysed the distributions of response latencies and noted optically induced changes (Supplementary Fig. 5). While too preliminary to allow for any biological conclusions, this data exemplifies the integration of optogenetic modulation with operant behavioural testing with pyControl. Note that, although a laser was used here, a low cost, open source, LED driver module has also been developed for pyControl which has been used successfully in other studies^11,28^.

## Discussion

We developed a novel open-source 5-choice operant box system characterized by low cost, rapid turn-over between subjects, and compatibility with implanted and tethered animals for physiological manipulations and measurements. The first two features facilitate training and testing of *large* cohorts in demanding operant tasks, while the latter supports state-of-the-art circuit neuroscience experiments in such behavioural paradigms. In our hands, assembly costs were around 700 EUR per complete setup unit, including operant box, outer cubicle and all peripherals; however, costs may vary depending on the sourcing of the plastic components, labour costs for building the outer cubicles, and economies of scale.

The system is one of several recent efforts from different labs to broaden the access to affordable operant box systems and to standardize experimental parameters and task structures by sharing task files^10^. While a detailed comparison of pyControl with the alternative open-source systems BPod and Bonsai is provided in our companion manuscript^10^, it is worthwhile drawing comparisons here between pyOS-5 and other systems that are more specifically designed to support operant-box experiments^3–6,8,9^. We designed our system to be fit for the specific purpose of 5-choice-based operant tasks and accessible to users without programming skills, while retaining a fully open-source framework, eliminating reliance on proprietary software. These priorities set our system apart from other open-source systems that employ generic microcontrollers, like Arduino^3,4^ or RasberryPi^4–6^, or generic task-scripting software designed to maximize wide-ranging applicability^7–9^. Instead, pyOS-5 combines a microcontroller with dedicated custom PCBs specifically designed for controlling operant behavioural experiments. Apart from simplifying the setting up of the system, this design also has the advantage of providing multiple input and output ports (e.g., dedicated BNCs, but also breakout lines from RJ45 sockets) that provide easy integration with electro- or opto-physiological data acquisition, or external stimulation devices like lasers, LEDs, speakers, or other microcontrollers. The commercial availability of pyControl electronic components at low cost through the Open-Ephys platform (https://open-ephys.org/pycontrol) alleviates the hurdle of organizing their fabrication based on the open-source design files while retaining cost-effectiveness. A further advantage of this design is the direct integration with the control software, including a dedicated GUI and a suit of ready-to-use task scripts, not offered by most alternative open-source systems.

In contrast to the 2-poke design of other poke hole-based open-source boxes^3,5^, a 5-poke wall can be used to assess a wider range of cognitive functions^2,12,13,19^ (see “Methods”) with a variety of task designs. These can be further enhanced by the auditory stimulation integrated in pyOS-5. A key advantage of a 5-poke over a classical 2-poke (or 2-lever) design of operant boxes are the high number of possible response configurations and the low (20%) chance level of correct responding. This is critical, for example, when assessing sustained attention in the 5-CSRTT to demand a spatially broad focus of attention and to prevent a strategy of obtaining sufficient rewards just from near-chance level performance^13^. Similarly in the 5-CSWMT this allows presentation of a large number of different two-hole or multi-hole choice configurations in the choice phase to prevent mediation strategies of positioning the body in one side of the chamber to solve the task without memory (discussed in more detail elsewhere^12,24^). Therefore, the wide applicability of the 5-poke design to assess a considerable number of cognitive functions is the key source of versatility offered by the pyOS-5 boxes. Several alternative open-source systems use touchscreens to present visual stimuli and register the animal’s choices^4–6^. Touchscreens certainly offer the advantage of a wider range of cognitive tasks, especially those relying on the visual discrimination of shapes as opposed to just spatial position and illumination, and they may potentially be more aligned with human assays^29,30^. The pyOS-5 system, in contrast, may be useful, if the classical poke-hole design is favoured given its wide application in the field over the recent two decades, and if the easy integration with physiological manipulation or recording is desired. Also, a large number of boxes (in our case 24, but there is no significant limitation for adding more) can be run simultaneously from a standard PC, without being limited by computing power. In summary, pyOS-5 offers great versatility for behavioural paradigms that rely on the well-established 5-poke design.

## Supplementary Information

Supplementary Information.

Supplementary Video 1.

Supplementary Video 2.

Supplementary Video 3.

## Data Availability

As stated in “Methods”, all design files and code necessary to build and operate the pyOS-5 system are available on GitHub (https://github.com/KaetzelLab/Operant-Box-Code; https://github.com/KaetzelLab/Operant-Box-Design-Files). All relevant information on pyControl is available on a dedicated website (https://pycontrol.readthedocs.io). Source data for the behavioural experiments can be obtained from the corresponding author (DK) upon reasonable request.
