# Supplementary material for: A low-cost open-source 5-choice operant box system optimized for electrophysiology and optophysiology in mice
Source: Sci Rep. 2021 Nov 15;11:22279. doi: 10.1038/s41598-021-01717-1 (PMC8593009; doi:10.1038/s41598-021-01717-1)
Supplement: Supplementary file 1 — Supplementary Information. [file 41598_2021_1717_MOESM1_ESM.pdf]

Supplementary Information

A low-cost open-source Python-based 5-choice operant box system optimized for electro- and opto-physiology in mice

Sampath K.T. Kapanaiah[1*], Bastiaan van der Veen[1], Daniel Strahnen[1], Thomas Akam[2,3], Dennis Kätzel[1,3*]

[1] Institute of Applied Physiology, Ulm University, Germany

[2] Department of Experimental Psychology, University of Oxford, UK

[3] Equal contribution

* Correspondence: dennis.kaetzel@uni-ulm.de (DK), sampath.kapanaiah@uni-ulm.de (SKTK)

Supplementary Figures

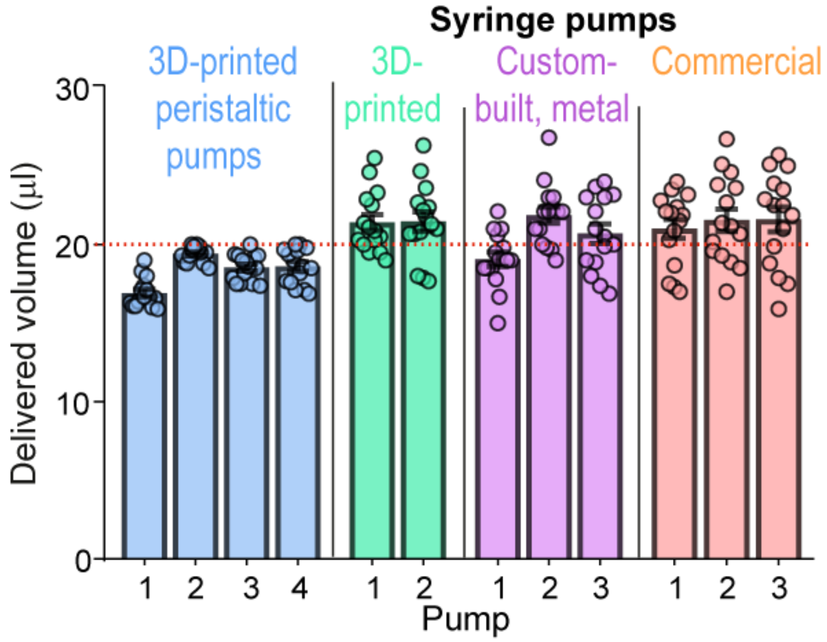

Supplementary Fig. 1. Delivery volumes of custom-made and commercial pumps. Volumes delivered by several randomly chosen individual peristaltic pumps (blue), custom-made 3D-printed (green) or metal (pink) syringe pumps, and commercially available syringe pumps (orange, Med Associates, Inc.) when the output volume was set to approximately 20 µl (red line) with 15 individual deliveries per volume and pump. Note that the setting of the output volume was identical for all individual pumps of a single kind, but differed between different kinds due to distinct coding of volumes by number of steps of the stepper motor or activated time for the commercial syringe pump (DC motor).

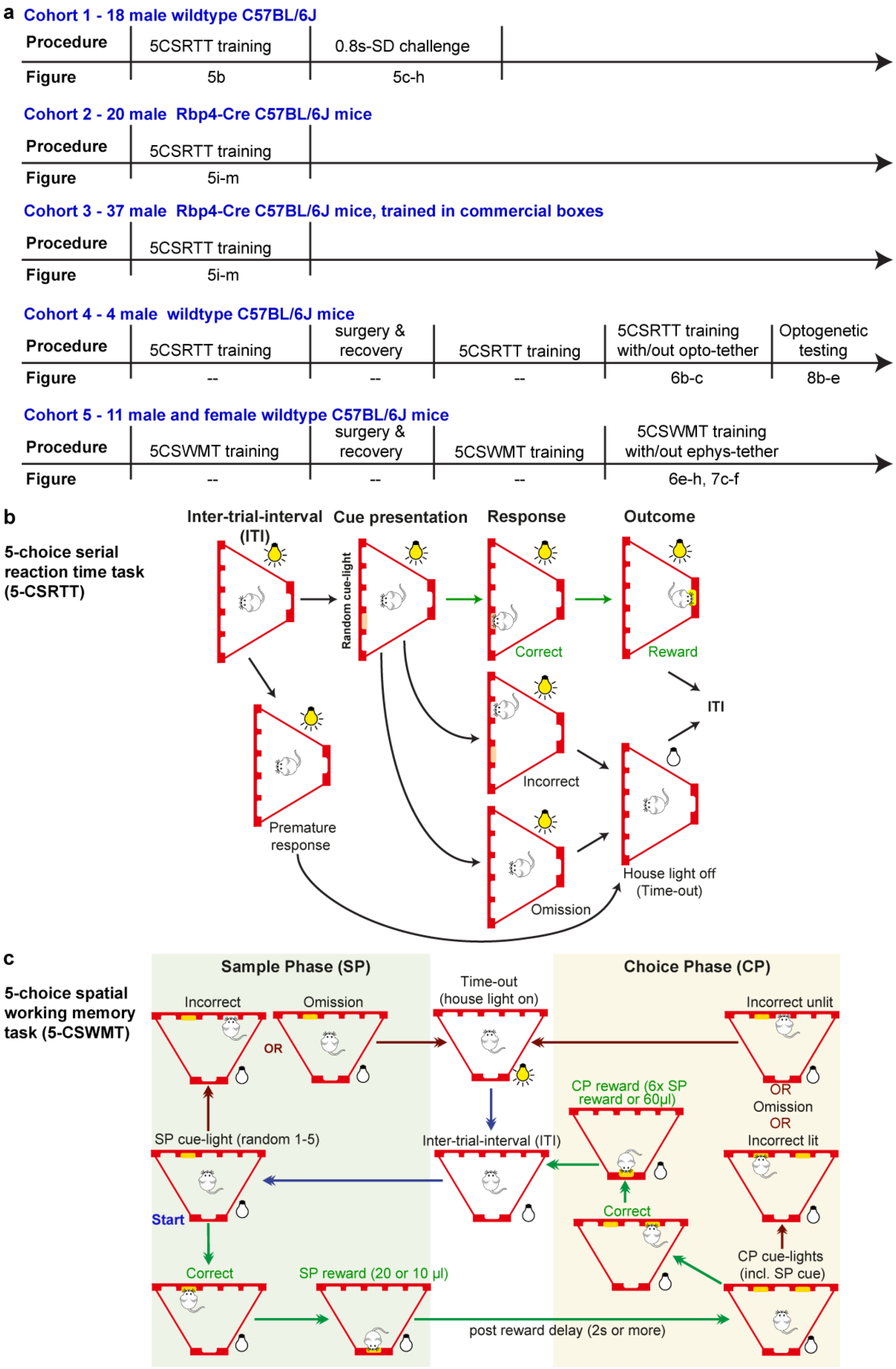

Supplementary Fig. 2. Temporal order of procedures and operant tasks. (**a**) Temporal order of training and testing in each of the 5 cohorts indicating at which stage the data

shown in the named main figure was obtained. (**b-c**) Task-flow of the 5-choice serial reaction time task (5-CSRTT; (b)) and the 5-choice spatial working memory task (5-CSWMT; (c)) as described in the Methods of the main manuscript. Cue holes to be poked are indicated by transient illumination. Note that the 5-CSRTT is conducted with an illuminated house-light by default (except for time-outs after erroneous choices) while the 5-CSWMT is conducted with the house-light switched off by default (except for time-outs after erroneous choices). Correct responses are rewarded by differing amounts of strawberry milk. The schemes were drawn in CorelDraw Graphics Suite 2021 (www.coreldraw.com).

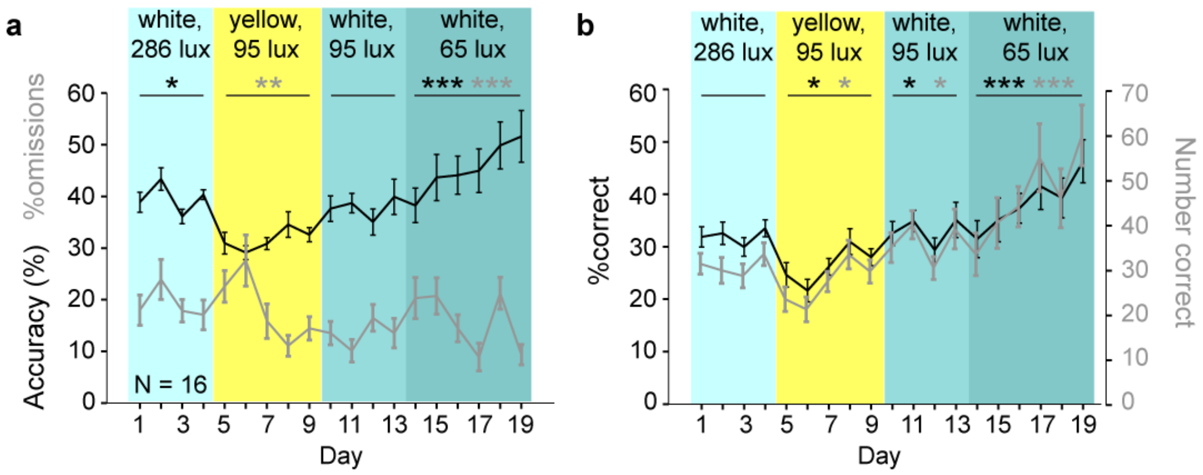

Supplementary Fig. 3. Initial training of cohort 1 in Stage 1 of the 5-CSRTT with different levels of illumination. (**a-b**) Attentional performance and task engagement measured by attentional accuracy (a, black), relative number of trials with omitted responses (a, grey), relative (b, black) and absolute (b, grey) numbers of achieved correct responses (and hence rewards) in a 30 min session with the utilised colour and brightness of the 5-poke indicator lights (indicated at top of graphs). Asterisks above horizontal lines indicate significant effects of *training day* (i.e. improvement or worsening of a given parameter over time) within one regime of light level as assessed by RM-ANOVA. The colour of the asterisk indicates the respective parameter to which it refers (*grey*, %omissions, a, or number corrects, b; *black*, accuracy, a, or %correct, b). Only 16 out of the 18 mice participated in the initial 14 days were light levels were changed, as the other two started later.

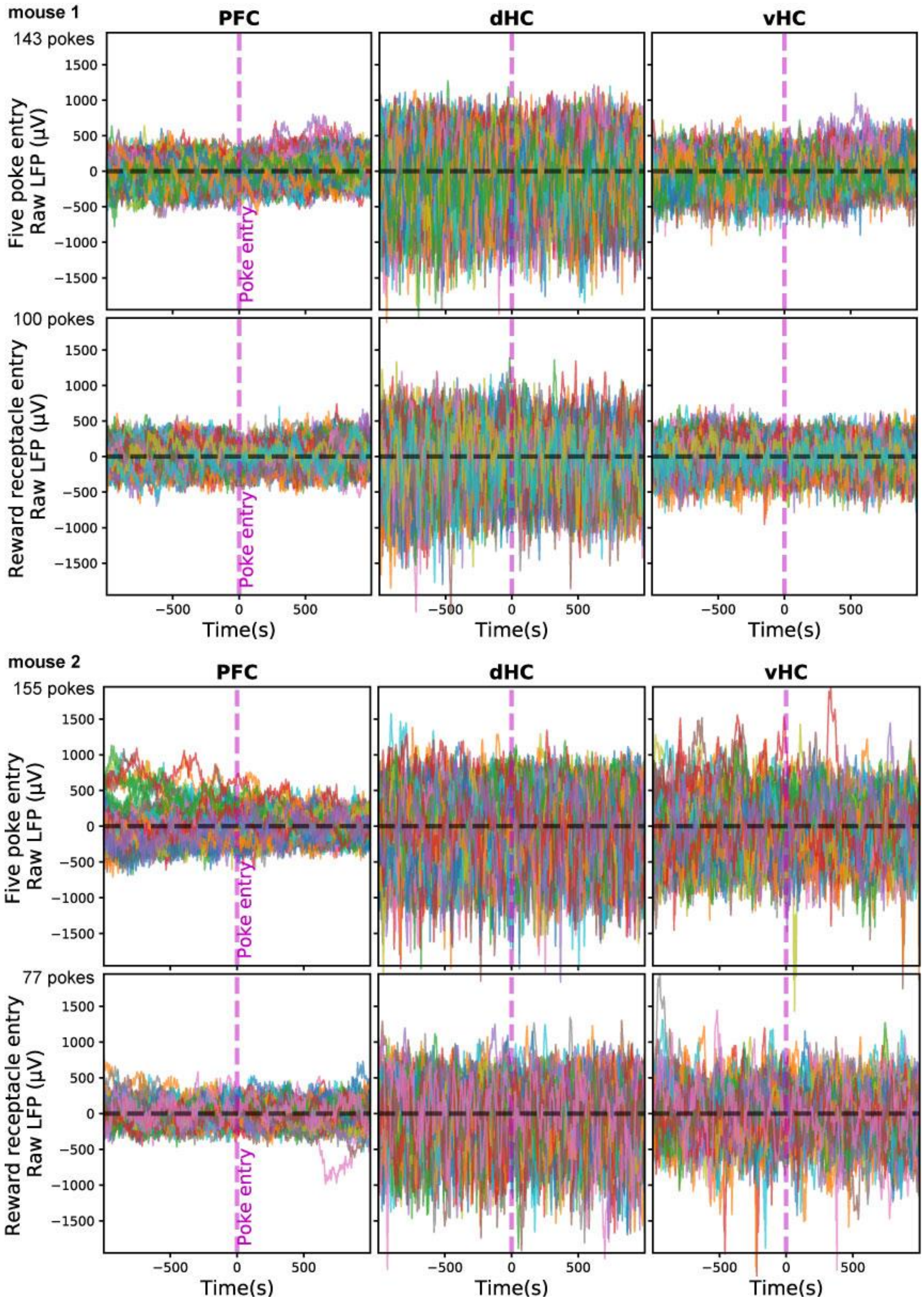

Supplementary Fig. 4. LFP recordings during poking. Individual raw traces recorded in the indicated brain regions of 2 mice (top and bottom) from cohort 5 around the time of poking into any of the 5-poke wall holes (decision pokes) or the reward receptacle (reward collection) coded by different colours.

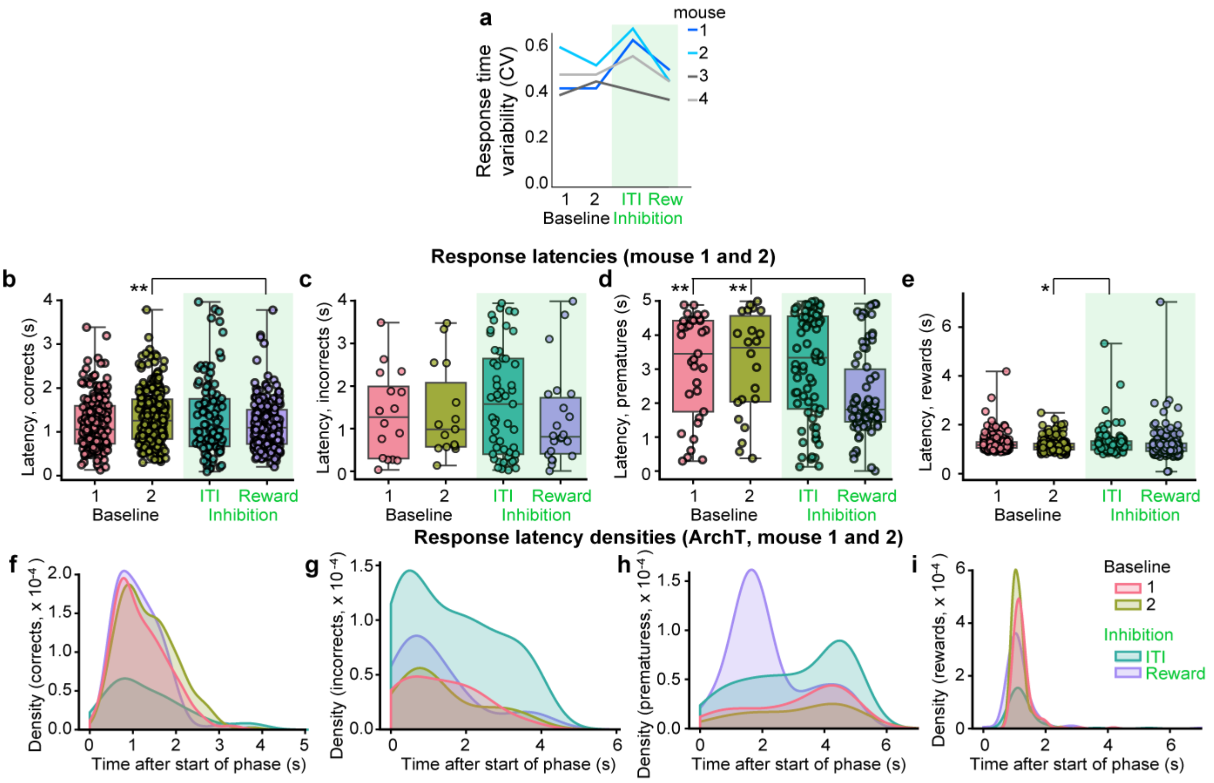

Supplementary Fig. 5. Changes in response latency distribution during optogenetic stimulation. (**a-b**) Variability of correct response latencies of each mouse (indicated by colour); measured as coefficient of variation, CV. Protocols with optical modulation indicated in green. Note that mice 1 and 2 were transduced with ArchT, while the remainder were transfected with Jaws. (**b-e**) Latencies of individual correct (b), incorrect (c), and premature responses (d) and of reward receptacle entries after correct responses (e) of ArchT-transfected mice (1 and 2) shown as dots for individual responses in each of the 4 sessions shown in Fig. 8b. Corresponding box-plots shown underneath. Asterisks indicate independent-samples *t*-tests. * $P < 0.05$, ** $P < 0.01$. (**f-i**) Same data as in (b-e) but shown as density distributions generated by convolution of the histogram with a Gaussian function in order to illustrate the latency distribution of each of the four response types in each of the four sessions.

Supplementary Tables

OEPS order#	PCB name	Remarks
py.002	Micropython breakout v.1.2	Main control board, we use a version with no parts on the front, BNCs mounted on the back to fix it to the outer box
py.025	Single poke v2.3	For reward receptacle, contains break-out connector to power the house-light (or other components) from the same board
py.026	Five poke v1.0	For 5-choice wall
py.022	Stepper driver v1.0 with Sparkfun Easydriver board (py.022)	For controlling the stepper motor of the reward delivery pump
py.050	Houselight v1.0 (3 LEDs)	To illuminate the interior of the outer and inner box with white light
py.024	Pass-through adapter v1.0	To break-out individual lines of the RJ45 cable, e.g. used to power the fan or camera, otherwise optional
py.020	Audio board v1.1	*Optional*: to deliver simple auditory stimuli (tones, white noise, clicks)
py.051	Speaker 4 Ω, 0-20 kHz, 4 W (Farnell # 1867858), connectorized	*Optional*: to deliver tones
py.031	Audio Player v1.0	*Optional*: to deliver arbitrary auditory stimuli from .wav files.
py.021	LED Driver v1.2	*Optional*: to power LEDs for optogenetic or optical stimulation
py. 052	HED Headstage	*Optional:* implantable LED for optogenetic stimulation

Supplementary Table 1: PyControl electronic components of the pyOS-5 box.

Item	Description	Order information
Colour/IR CCTV camera	Pinhole Kamera 205-IR-L, 5 MP	Kobert-Goods UG, Melsungen, G (or amazon.com)
Stepper motor for peristaltic or syringe pump	Nema17, 2-phase, 4-wire, 1.7 A current drawn per phase, 0.4 Nm torque, 1.8° step angle;	part# 17HS4401 on amazon.com
Cable to connect motor to control board	NEMA17 stepper motor cable, 6-pin JST to 4-pin Dupont plug, 1 m	SIENOC 3D-AC27* from amazon.com
Fan	Axial Fan, 12 V DC, 40 mm	Part# 2543656, Farnell
AC/DC power supply	5 W, 12 V, 420 mA	Part# 2451883, Farnell
RJ45 cables	Cat5e, Connecting breakout-board with other peripherals, 0.15, 0.25 and 0.5 m, 1 m (white for single-poke)	Any electronics supplies
USB2.0 cable	USB 2.0 male A > male micro-B; 1.8 m or 3 m	Any electronics supplies
USB hub	USB 2.0 hub, 13-port (for up to 13 boxes)	LOGILINK UA0126; any electronics supplies

Supplementary Table 2. Additional electronic components of the pyOS-5 box.

	**Parameters of training**			**Criteria for stage transition (2 consecutive days)**			
**Stage**	SD (s)	LH (s)	ITI (s)	# correct	% correct	% accuracy	%omissions
**1**	20	22	2	>= 30	>= 40	-	-
**2**	8	10	2	>= 40	>= 50	-	-
**3**	8	10	5			>= 80	<= 50
**4**	4	6	5			>= 80	<= 50
**5**	2	4	5			>= 80	<= 50
**Challenge**							
6	0.8	3	5	Attention challenge			

Supplementary Table 3. 5-CSRTT Training and challenge stages. The parameters stimulus duration (SD), limited hold (LH) and intertrial-interval (ITI, waiting time before stimulus) are listed for each of the 5 training stages (1-5; stage 5 constitutes the baseline stage for further experiments) and the subsequent challenge protocol on which performance was tested for one day. The performance criteria which had to be met by an animal on two consecutive days in order to move to the next training or the test stage are listed on the right. See main text for the definition of these performance parameters. #correct, total number of correct responses made during a 30 min session.

	**Parameters of training and baseline stages**					
Stage	SP-SD, s	CP-SD, s	Pre-delay, s	Post-delay, s	Reward (µl)	CP configurations
1	20	20	0	2	20	1-3, 2-4, 3-5
2	20	20	0	2	10	1-3, 2-4, 3-5
3	20	20	0	2	0	1-2, 2-3, 3-4, 4-5
4	20	20	0	2	10	1-2, 1-3, 1-4, 2-3, 2-4, 2-5, 3-4, 3-5, 4-5
5	10	5	0	2	10	1-2, 1-3, 1-4, 2-3, 2-4, 2-5, 3-4, 3-5, 4-5

Supplementary Table 4. 5-CSWM task training stages. Note that, on *all* stages, the *post-delay* is 2 s, the *CP reward* is 60 µl, the *limited hold time* - that is the time in which a response is registered - exceeds the SD by 1 s, the *ITI* is 5 s, and the time-out duration after incorrect responses or omissions is *5 s*. The CP configurations indicate the two holes of the 5-choice wall that can be illuminated; note that the actual configurations when taking into account the correct hole is double than what is stated (i.e., 2-4 is different from 4-2, which is not listed). Stage 4 served a pre-surgery baseline and post-surgery training stage without tether. Stage 5 is the baseline stage on which mice were trained immediately before and after being tethered for the first time (data shown in Fig. 6).

Supplementary Videos

Supplementary Video 1. Animation of the pyOS-5 operant box assembly (version with snap-lock instead of magnet for the door).

Supplementary Video 2. Execution of a correct poke and reward collection in the 5-CSRTT.

Supplementary Video 3. Continuous performance in the 5-CSRTT with mounted headstage and tether.

Supplementary Methods

Supplementary Construction Guides

1. Construction guide for pyOS-5 boxes

1.1 Layout of all components of the operant box (except for M3 screws and door magnet). Components in the image appear approximately from bottom to top in the left-to-right order and, regarding poke walls, from outside to inside in the top-to-bottom order.

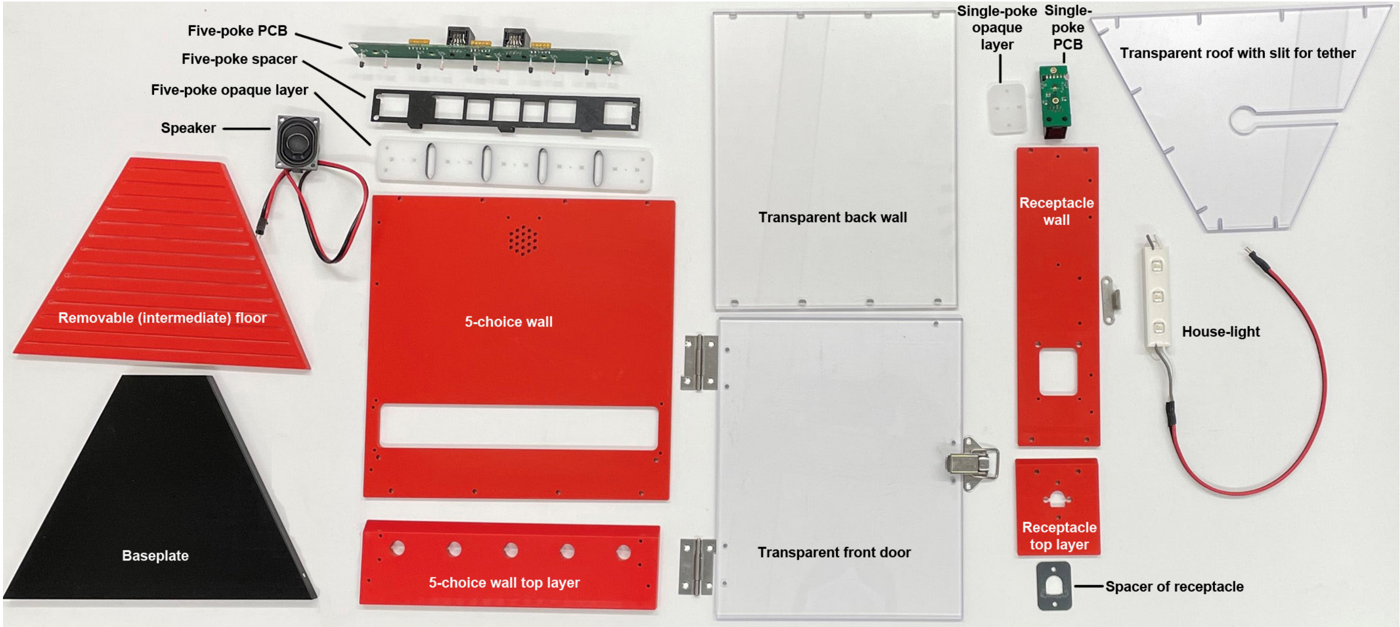

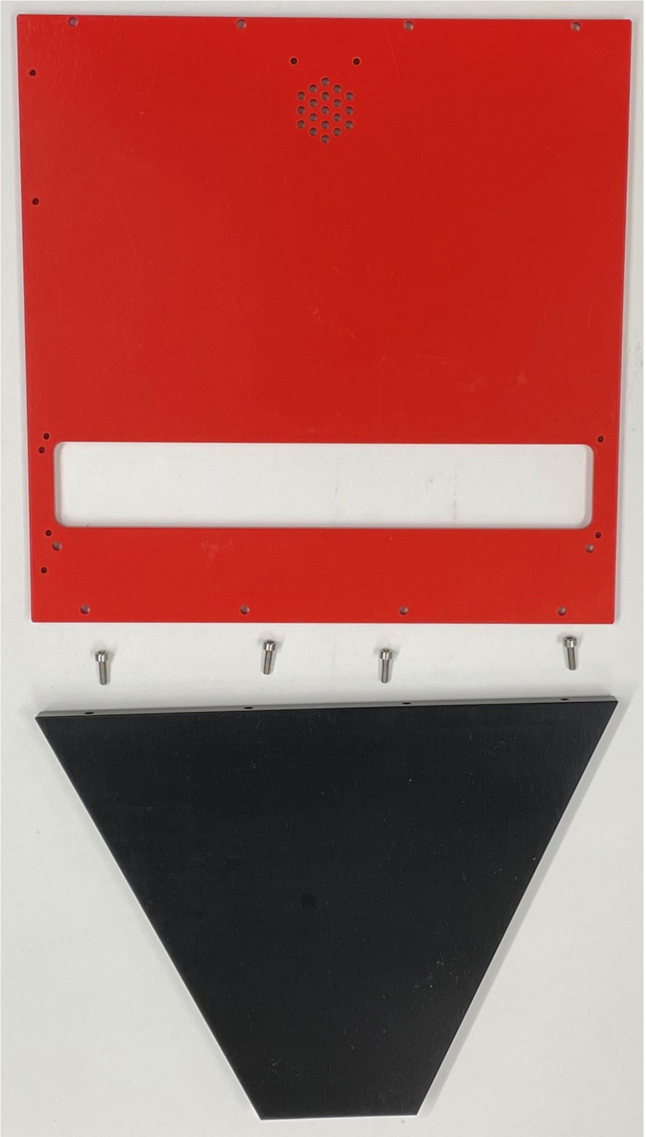

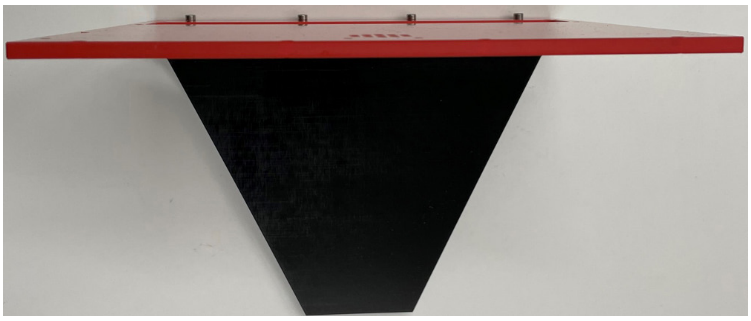

1.2 Screw **baseplate** (lower floor) to **5-choice wall** using 10 mm M3 screws (left: components; right: final assembly viewed from top).

1.3 Assembly of components of the 5-choice wall. Right: All required components of the inset of the 5-choice well, incl. 16 mm M3 screw, in order of assembly from outside (top) to the inside (bottom) of the box.

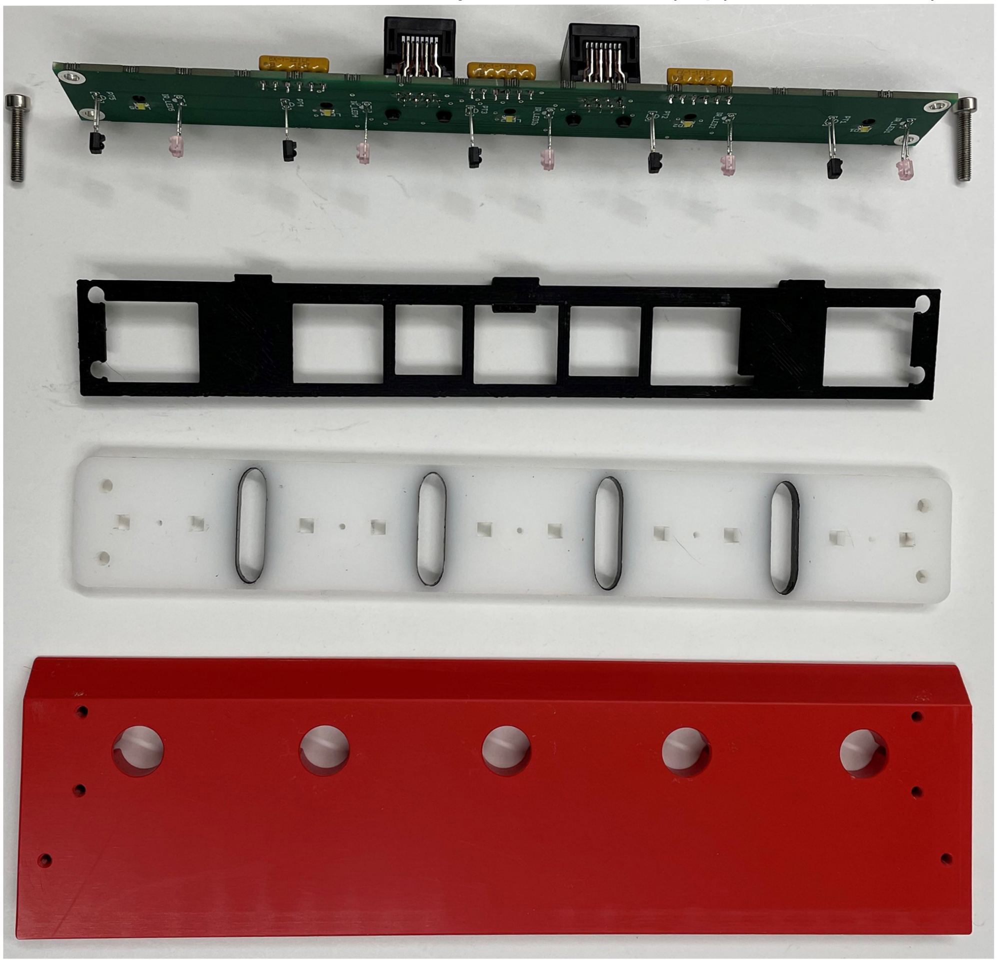

Note that the inside rim of the elongated slits of the 5mm **opaque spacer layer** are painted with black colour (marker pen) to aid absorption of light from neighbouring indicator light of the 5-poke PCB. To increase opacity, stripes of white tape may be added to this layer.

All 6 holes of the red top layer of the 5-choice wall have internal M3 threads. The upper 4 holes take the screws that hold the 5-poke-PCB and all intermediate layers together (we usually use only 2 screws, even though 4 can be used), the lower 2 holes are for mouting the hole assembly to the 5-poke wall from step 2.

1.3a Attach the 3D-printed black spacer layer to the 5-poke PCB. (Photos show the same state from two angles.)

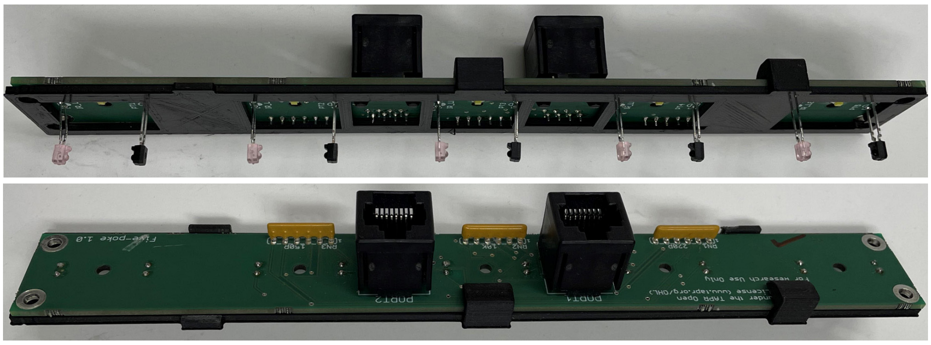

1.3b Assemble **PCB with spacer** and **5mm opaque spacer layer**, and fix the assembly in place by screwing it to the inner 5-poke layer with M3-screws. The photos show the same state from three angles; note the order of layers in the top panel, the fixation with 2 screws in the middle panel, and the lack of screws or nuts on the inside layer (due to internal threads) in the bottom panel.

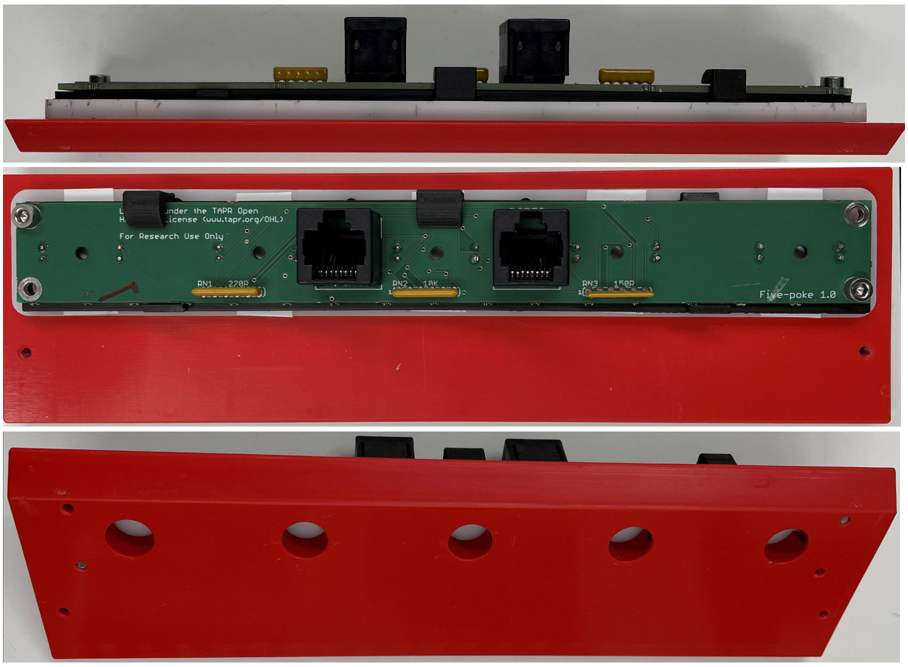

1.3c Attach assembled layers to 5-poke wall using two 10 mm M3 screws inserted from the back (outside, top right panel). The layer assembly is brought to the wall from the inside (bottom panel). Note the lack of screws or nuts on the inside layer (due to internal threads) and the 2mm diameter through-holes penetrating all layers to allow provision of reward, if desired, in the bottom panel.

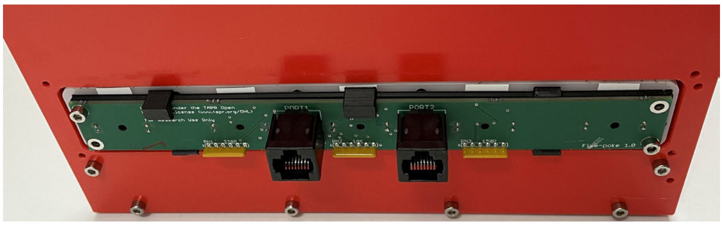

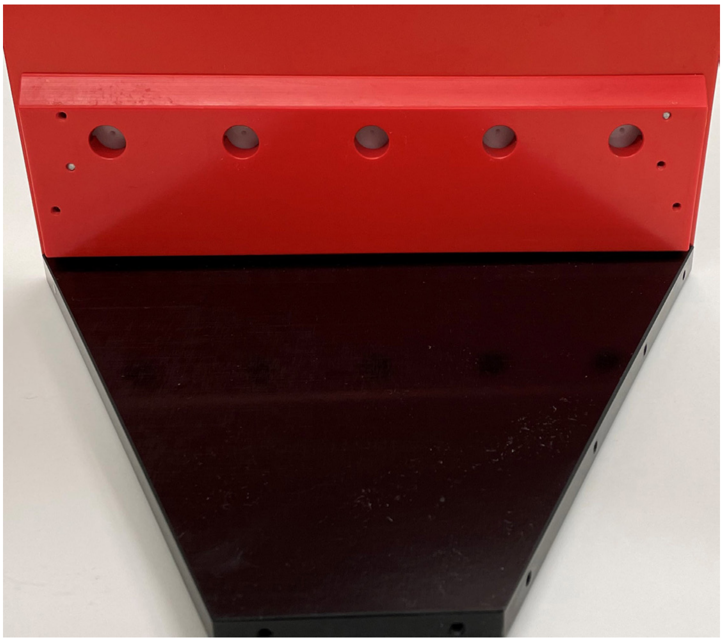

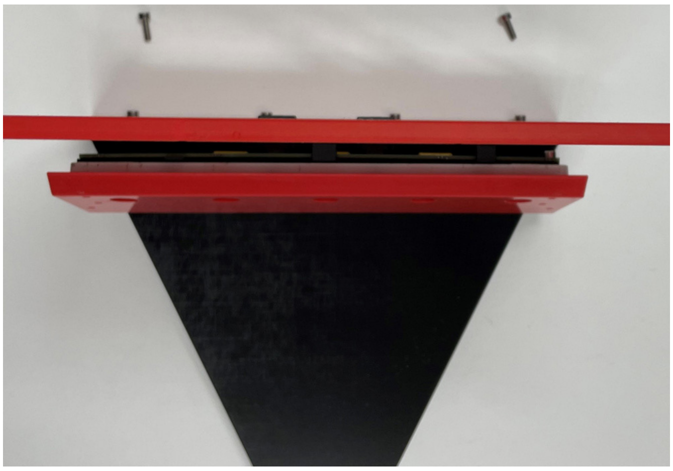

1.4 Assembly of components of the receptacle-wall. Display of components of the receptacle wall (left) and their layered assembly order (right).

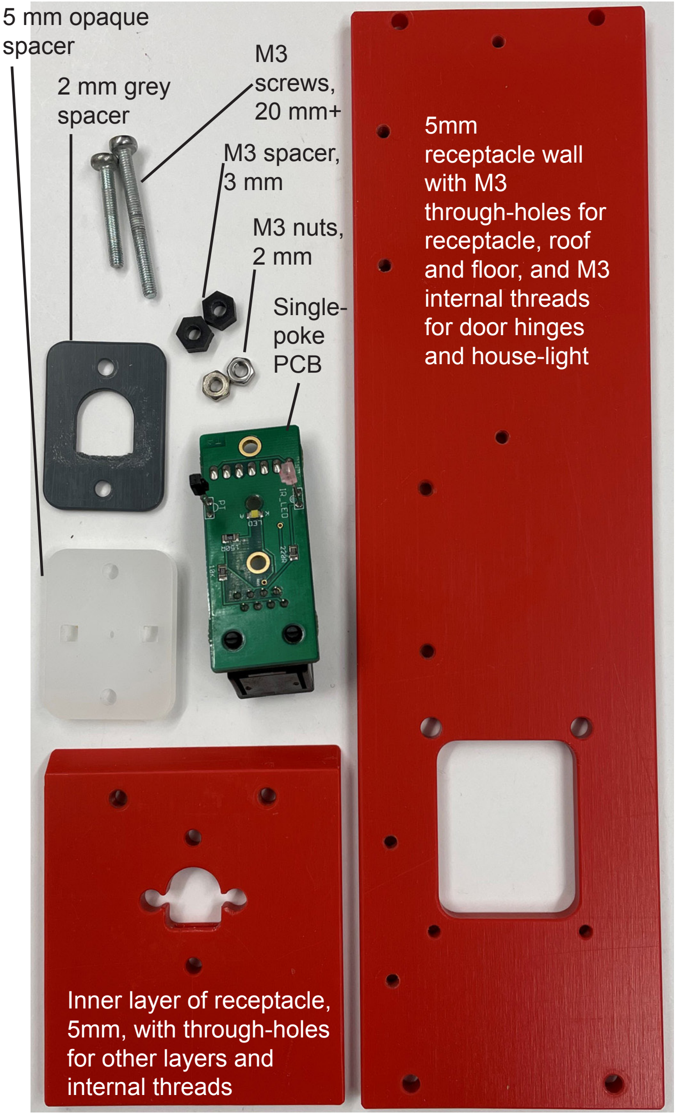

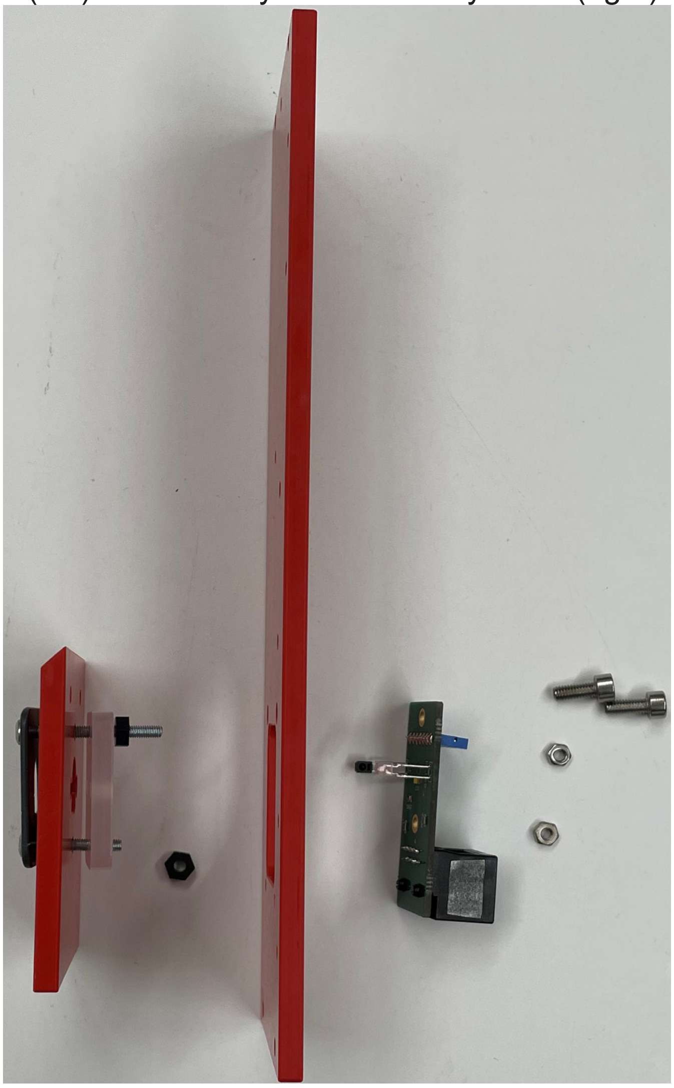

1.4a String together inner (grey) **spacer layer**, **inner receptacle layer** (red) and **opaque spacer** using flat-headed M3 screws and insert into **receptacle wall** (red).

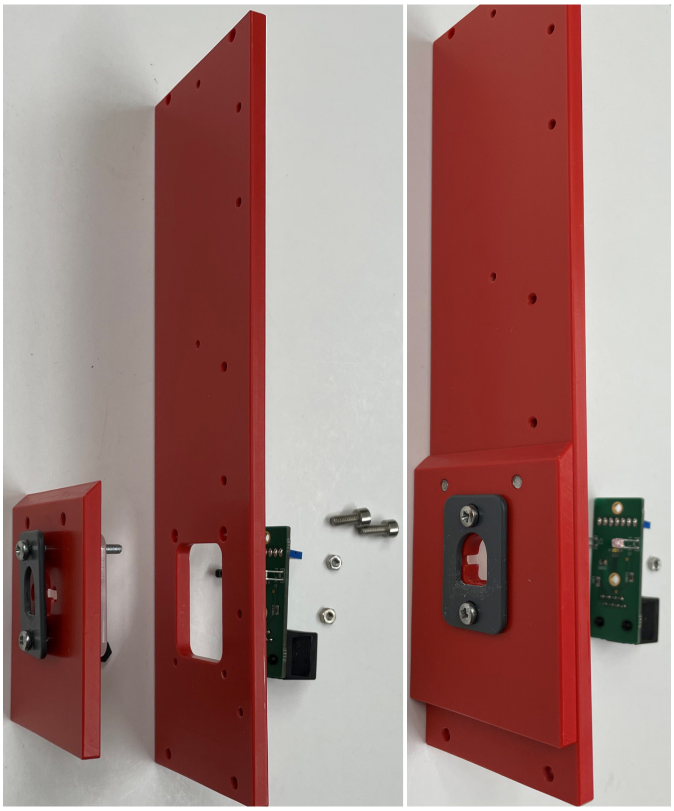

4b. Add 3mm M3 spacer nuts (black), **single-poke PCB** and M3 nuts.

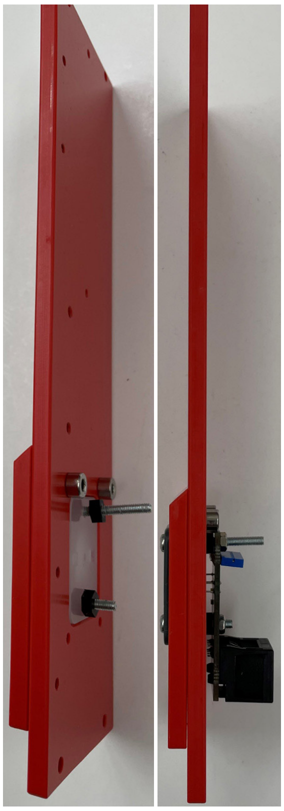

4c. Fasten **layer assembly** to **receptacle wall** with 10mm M3 screws screwed into internal threads of inner layer.

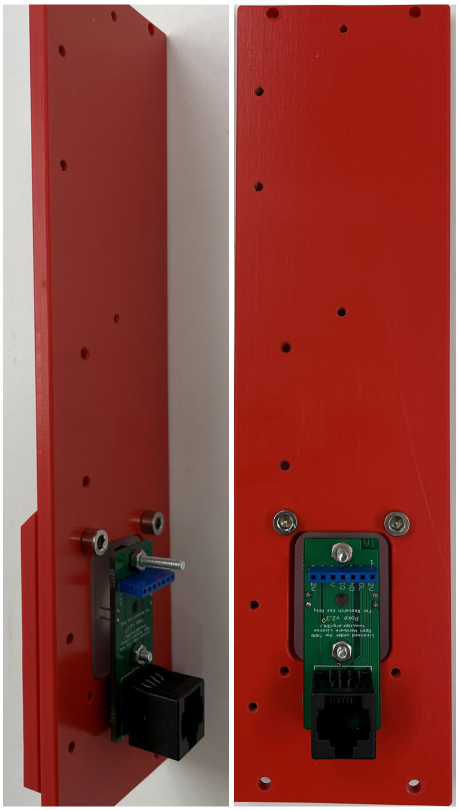

1.4d Attach **house-light** (LED array) to outside receptacle wall using 6 mm M3 screws. Connect cable to blue break-out connector as shown.

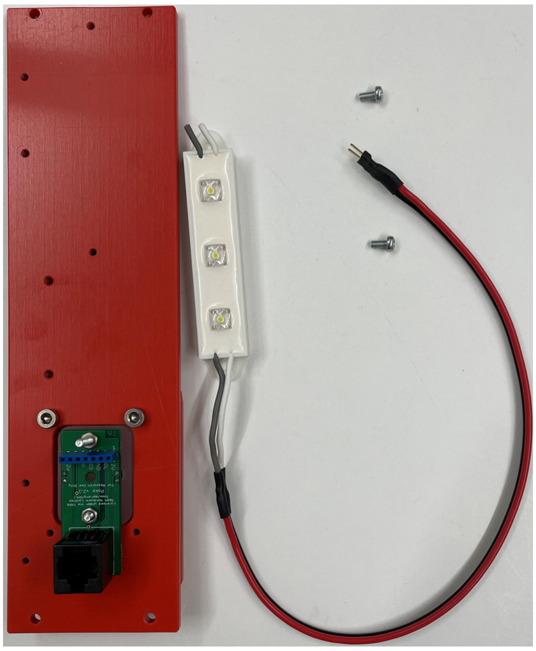

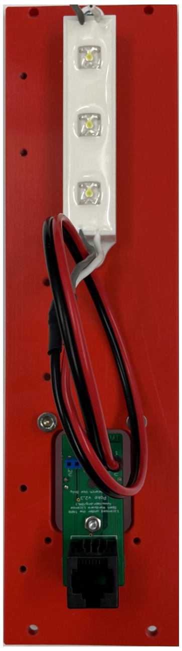

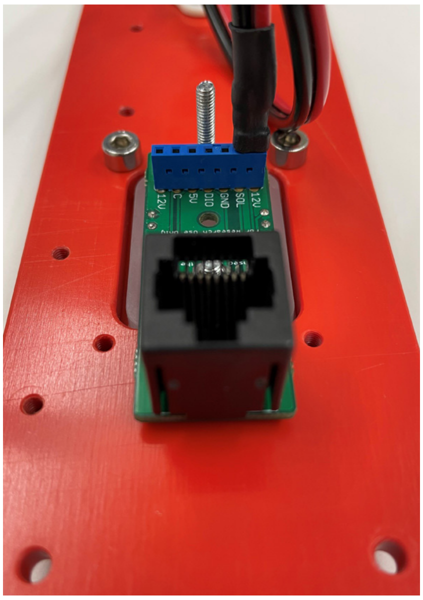

1.5a Assembly of front door. Metal hinges are screwed to internal threads in 5mm thick Perspex front door using 6 mm M3 screws. Magnetic rod is inserted into lower left hole and serves simultaneously as door lock and handle to open the door. Left, partially assembled; right fully assembled.

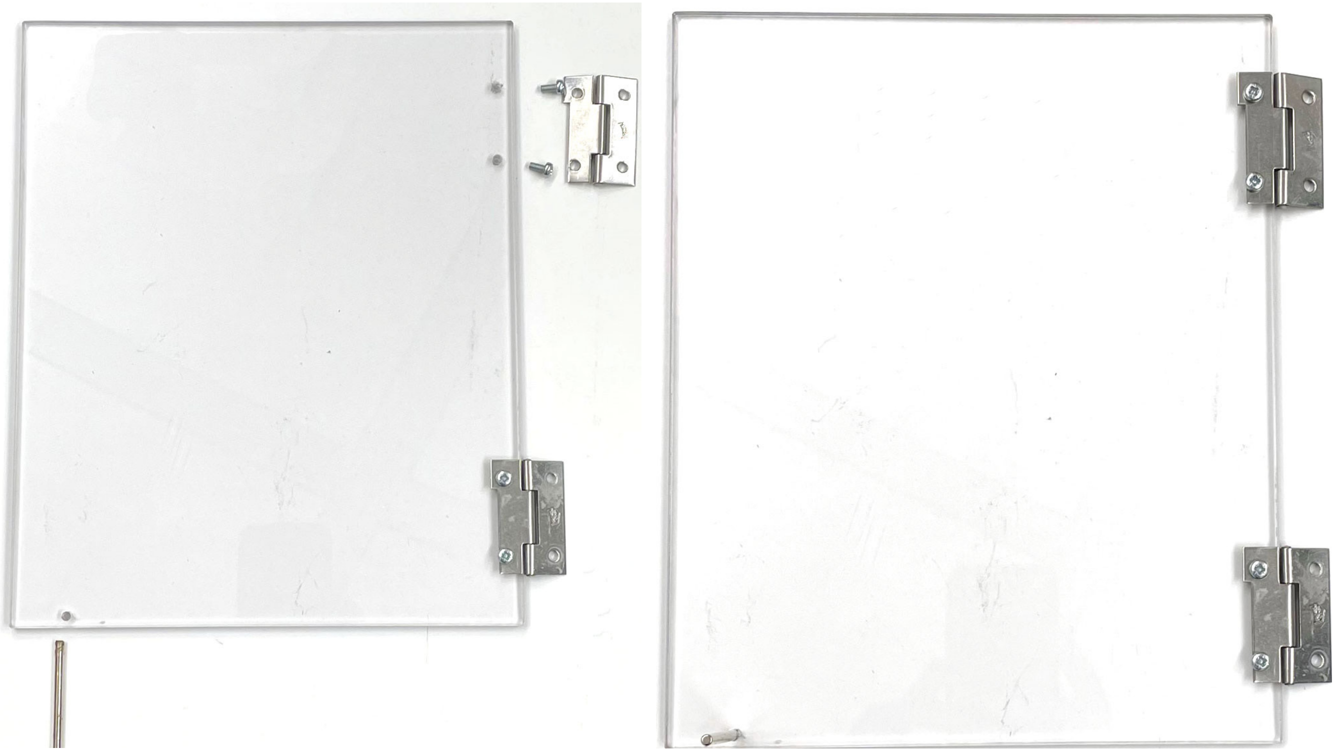

1.5b Connect front door to receptacle wall. Metal hinges are screwed to internal threads in 5mm thick red receptacle wall using 6 mm M3 screws. Left, partially assembled; right, fully assembled.

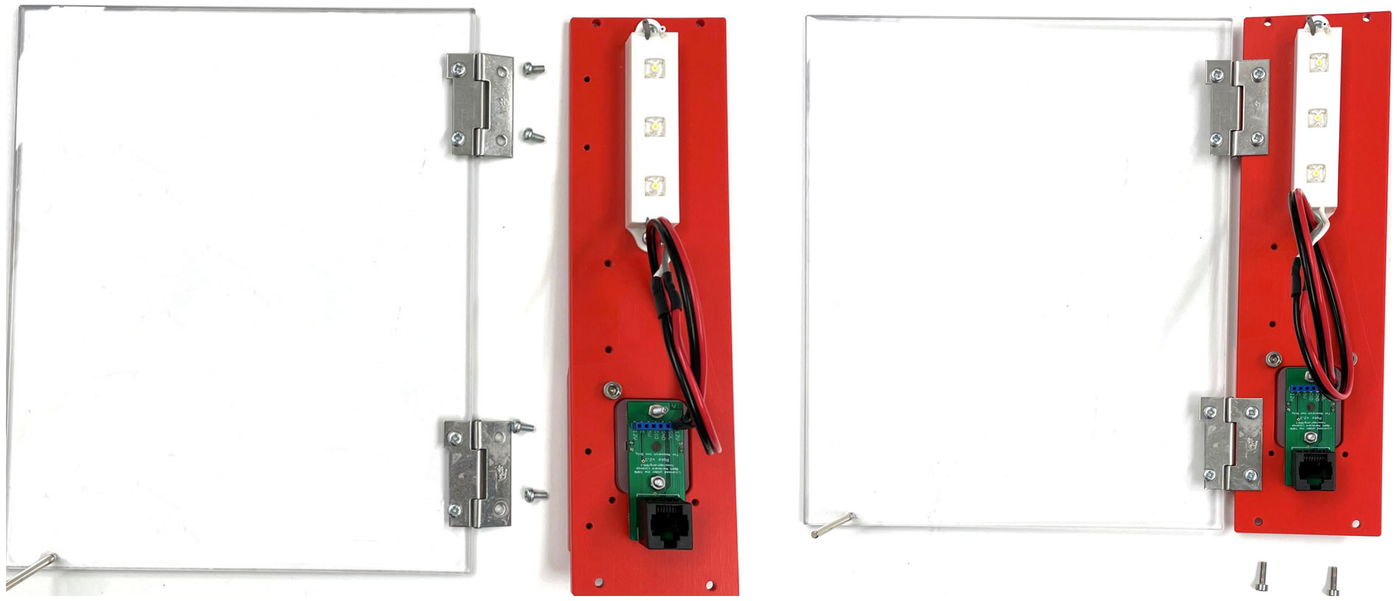

NOTE: this guide shows the box design with the door opening on the side of the 5-choice-wall and kept close by a magnet. For a version with the door opening at the receptacle side and closed by a snap look refer to Supplementary Video 1 and the design files on https://github.com/KaetzelLab/Operant-Box-Design-Files/tree/main/Additional%20 Design%20files ; the assembly is virtually identical except for a 180° inversion of the door, use of the snap lock and use of different holes at the 5-choice vs. receptacle wall. Both versions work well in practice; the snap lock provides a tighter closing of the door, which is why design files are provided for this version.

1.6 Connection of all walls. Screw transparent back wall to black floor with 10 mm M3 screws, then screw receptacle wall with front door to floor with 10 mm M3 screws.

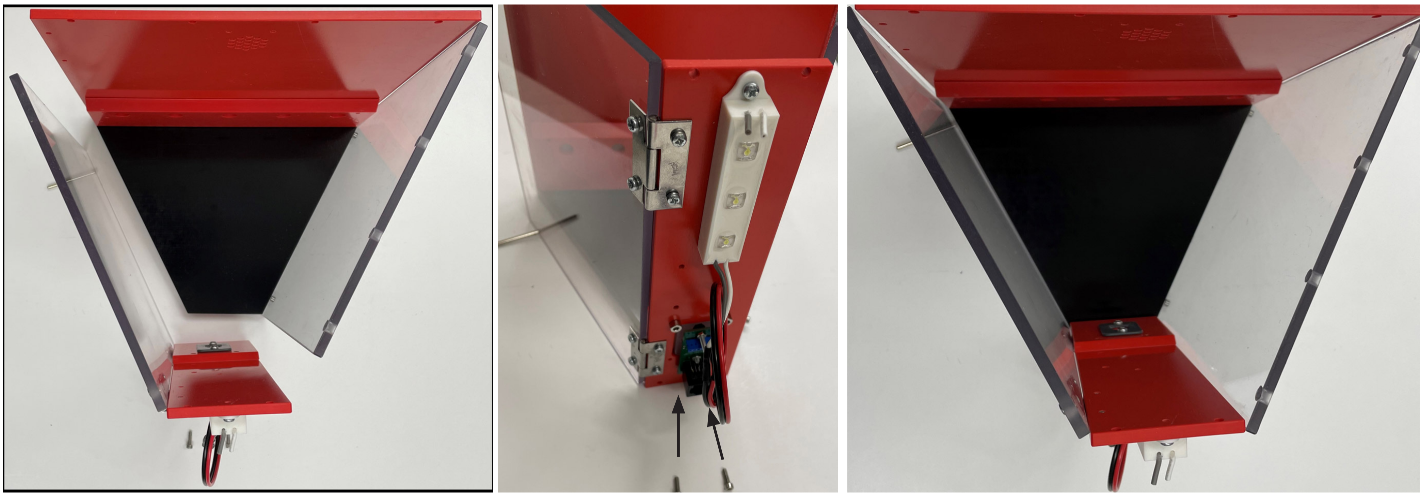

1.7a Screw roof to 3 walls with 10 mm M3 screws using internal threads in roof.

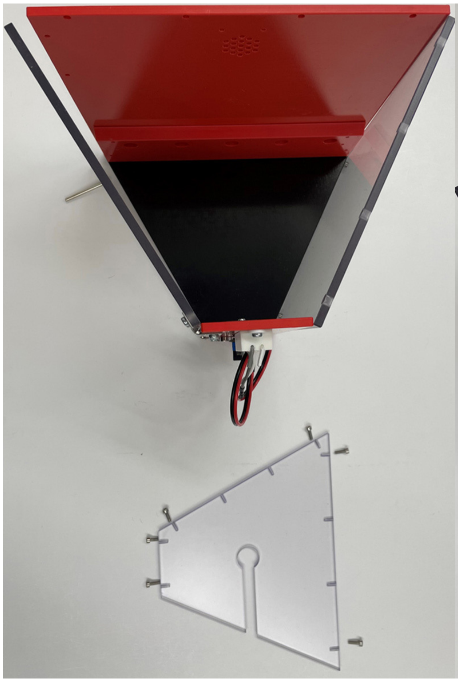

1.7b Screw speaker to 5-poke wall with 6 mm M3 screws using internal threads in wall.

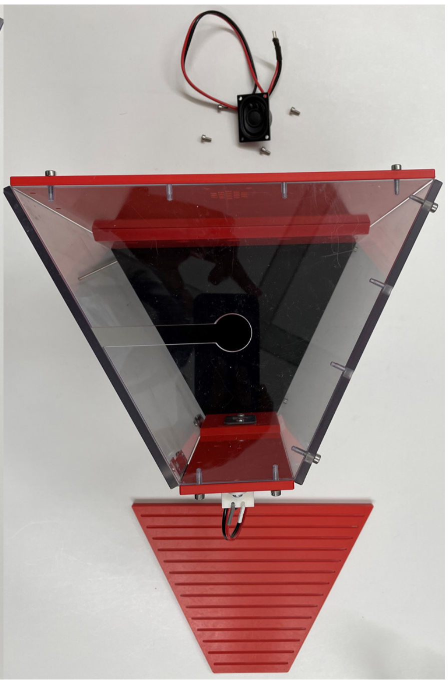

1.7c Add intermediate (re-movable) red floor with 45° grooves..

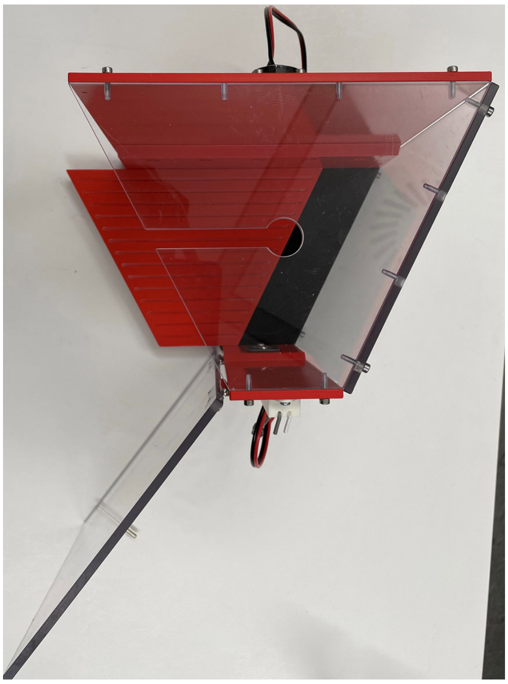

1.8 Connect 1 m ethernet cable to single-poke RJ-port and 0.25 m ethernet cable to 5-poke RJ-port.

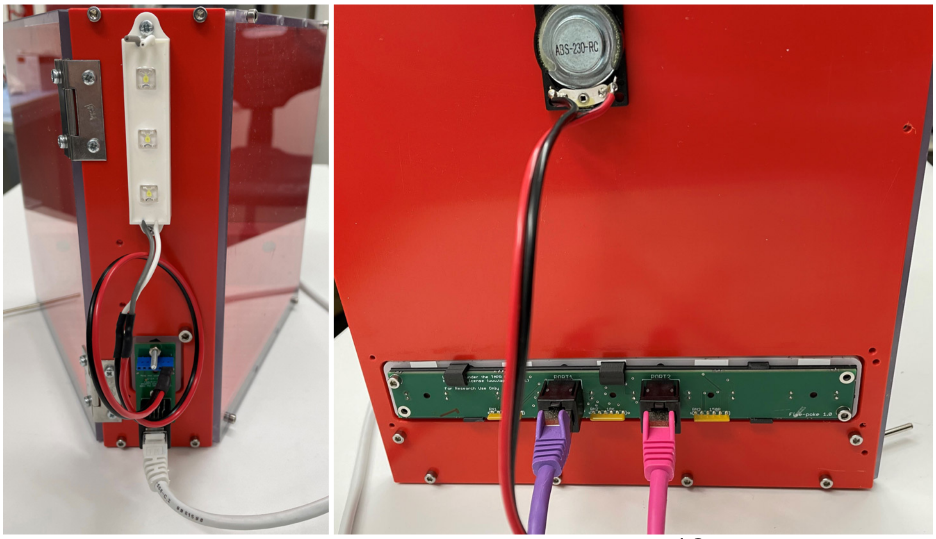

DONE!

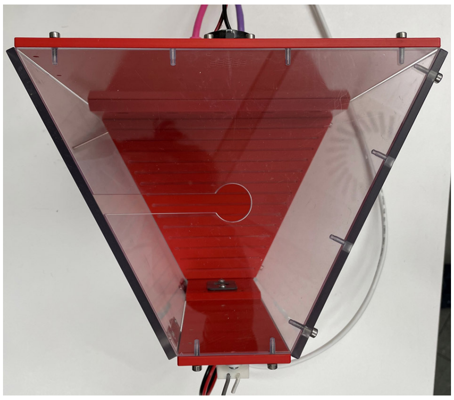

1.9 Overview of parts lists for operant box (number per box; excluding outer cubicle)

1.9.1 PVC and perspex parts (obtained from mechanical workshop, CNC-machined according to design files supplied on https://github.com/KaetzelLab/Operant-Box-Design-Files), 1 per box:

Part	Colour	Material, mm	PDF design file
5-choice wall	red	PVC, 5	18_0007_003
5-choice inner layer	red	PVC, 8	18_0007_014
Receptacle wall	red	PVC, 5	18_0007_002
Receptacle inner layer	red	PVC, 5	18_0007_012
Receptacle spacer	grey (any)	PVC, 2	18_0007_013
Baseplate (ground floor)	black (any)	PVC, 10	18_0007_001
Removable (intermediate) floor	red	PVC, 8	18_0007_015
Back wall	transparent	Perspex, 5	18_0007_004
Front door (*version with snap-lock, opening on receptacle side)	transparent	Perspex, 5	18_0007_005 *
Roof	transparent	Perspex, 5	18_0007_008

1.9.2 Additional plastic parts (1 per box)

Part description, material, thickness	Potential source	Design files
5-poke opal layer, opal030* acrylic, 5mm	CutLaserCut UK, Ltd.	5mm_opal030_acrylic_200_x_30mm 5-poke
single-poke opal layer, opal030* acrylic, 5mm	CutLaserCut UK, Ltd.	5mm_opal030_acrylic_26_x_33mm receptacle poke
Black 5-poke spacer layer	3D-printing	five_poke Layer.stl

* Opal030, when leaving one original protective layer on the acrylic and adding white lab tape on the other, yields ca. 65 lux output. Acrylic boards with lower transmissivity (Opal050 or Opal070) may be used to get lower light levels.

1.9.3 Screws and additional non-electronic items (not including parts for pump):

Item	Q'ty	Potential source (order#)	Remarks
M3 screw, 6 mm	16	Farnell (1419600 / M3 6 SO12CS S100, or 1419986)	hex or Phillips socket
M3 screw, 10 mm	26	Farnell (1419920 / M3 10 SO12CS Z100)	hex or Phillips socket
M3 screw, 16 mm	2	Farnell (1419922 / M3 16 SO12CS Z100, or 1420393)	hex or Phillips socket
M3 screw, 20 mm	2	Farnell (1419988 / M3 20 PRSTMC Z100)	flat Phillips head
M3 spacer, 3 mm	2	Farnell (359-154 or 3754457)	
M3 nut, 2 mm	2	Farnell (1420788 or M3-HFA2-S100)	
2 mm x 40 mm tube	1	Misumi (SPLS2-40)	ID 1 mm, reward tube
Magnet, 1/8" x 1/8" cylinder	1	KJ Magnetics (D22)	Door closing magnet
Magnet, 1/8" x 1 ½" cylinder	1	KJ Magnetics (D2X8)	Door closing magnet/handle

1.9.4 Cabling and electronic items (quantity per box; without BNC cables & pyControl PCBs)

Item, description	Q'ty	Potential item/source (order#)	Remarks
Ethernet cable (Cat5e, RJ45), diff. colours, 25 cm	2	various	connecting 5-poke PCB
Ethernet cable (Cat5e, RJ45), diff. colours, 15 cm	2	various	connecting audio & stepper PCBs
Ethernet cable (Cat5e, RJ45), 25 or 50 cm	1	various	connecting breakout board for fan
Ethernet cable (Cat5e, RJ45), white, 1 m	1	various	connecting reward receptacle PCB
USB2.0 male A > mal micro B, e.g. 2-5 m	1	various	connecting pyControl PCB to PC
USB2.0 hub	1	e.g. LOGILINK UA0126 13-hub	connecting multiple boxes to PC
Axial Fan, 12 V DC, 40 x 10 mm, 8 cu. ft/min	1	Farnell (2543656)	mounted to outer cubicle
DC Power supply 5 W, 12 V, 420 mA	2	Farnell (2451883)	2nd one for camera optional
Speaker, 4 W, 4 Ohm, 0-20 kHz, ABS-230-RC	1	Farnell (1867858)	or bought connectorized from OEPS
NEMA17 stepper motor cable, 6-pin JST to 4-pin Dupont plug, 1 m	1	e.g. SIENOC 3D-AC27	stepper-motor-driver PCB to motor
Nema17 stepper motor, 2-phase, 4-wire, 1.7 A drawn/phase, 0.4 Nm torque, 1.8° step angle	1	amazon.com (17HS4401)	for peristaltic or syringe pump
Pinhole Kamera 205-IR-L, 5 MP	1	Kobert-Goods UG Melsungen	Colour/IR CCTV camera

2. Construction guide for 3D-printed peristaltic pumps

All 3D printing files can be downloaded from **GitHub** https://github.com/KaetzelLab/Operant-Box-Design-Files/tree/main/Peristaltic%20Pump. The achievable volume and its consistency depends on the 3D printing resolution (not >0.2 mm) and assembly of the pump hence it is advisable to print at 0.1 mm resolution and check delivered volumes for all assembled pump individually. Printing material could be anything ranging from PETG or PLA or ABS, pumps used in the manuscript were printed using polylactic acids (PLA, biodegradable plastic).

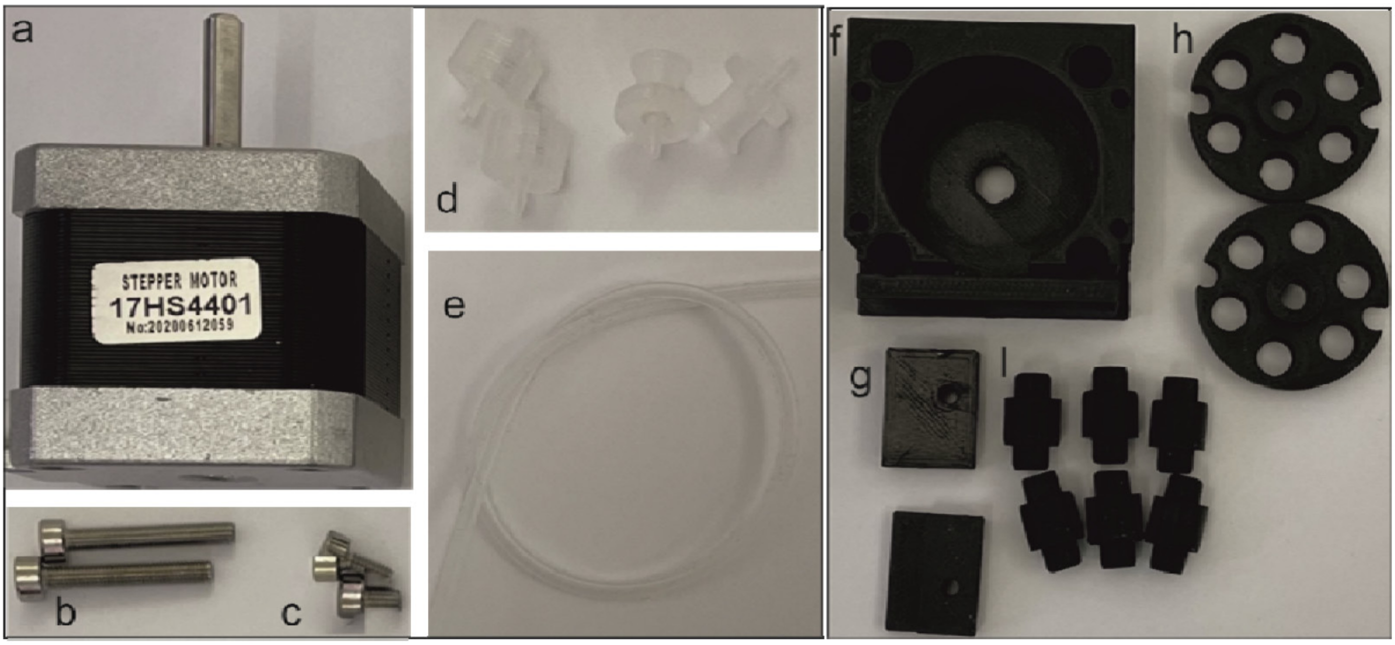

2.1 Images (top) and list (right) of **individual components** of 3D printed peristaltic pumps

Item, description	Q'ty	stl file / part name / potential supplier (order#)
a, stepper motor	1	Nema-17 Stepper motor; e.g. Amazon (17HS4401)
b, screws	2	M3*20 mm scews with hex socket head, e.g. Farnell (1419947)
c, screws	2	M3*6 mm scews with hex socket head, e.g. Farnell (1419600)
d, luer lock	2	1.6mm ID luer hose connector; e.g. Carl Roth (CT58.1 & CT62.1)
e, Reward tube	1	Silicon tube, 1.60 mm ID, 3.20 mm OD; e.g. VWR (228-1471)
f, housing structure	1	Bottom.stl
g , tube end adapters	2	Adaptor.stl
h, bearing spinner	2	Wheel.stl
i, rollers	6	Bearings.stl

2.2 Assembly of 3D-printed parts. Mount 3D-printed bottom to stepper motor using M3*6mm and M3*20mm Socket Head Cap Screws (**a**) and arrange 6 bearings between two wheels as shown in (**b**). Fix the bearing wheels inside bottom-stepper assembly (**c**).

Insert the adapter to one end of the tube and make incision on other end of the tube (helps to pass through adapter shallow hole; **d**), and pass the tube through adapters facing each other, to look like (**e**).

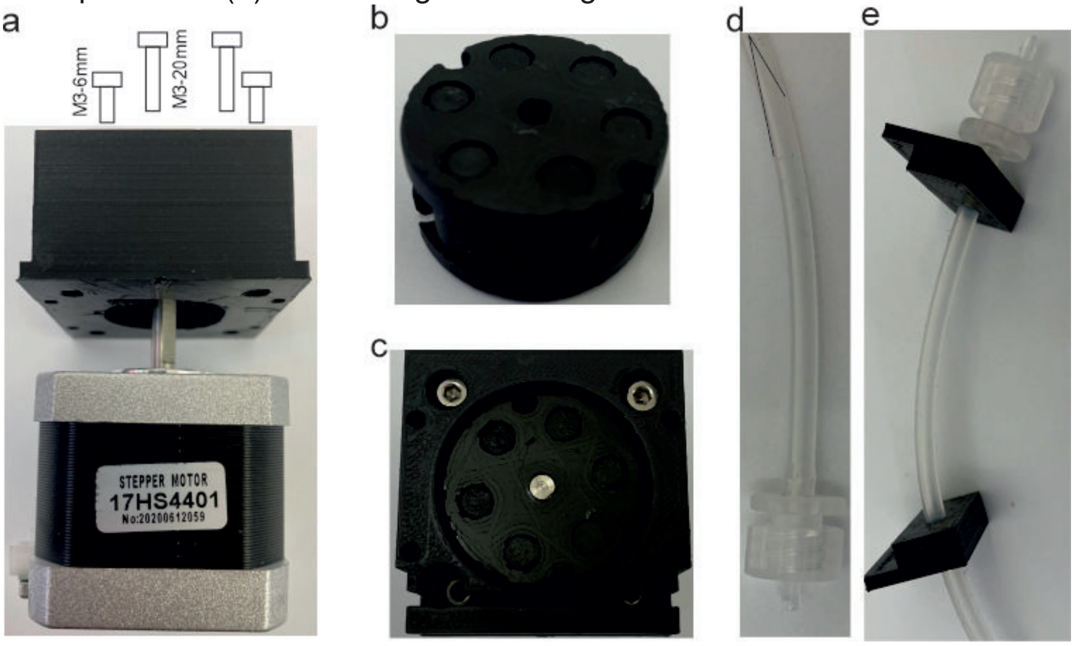

2.3 Insertion of tubing. Pass **tubing** one full rotation between rollers and the housing's inner wall by slowly rotating **wheel** one full rotation as shown in the (**a**). Secure tightly by pulling tube on the other end of the pump to obtain the final arrangement shown in (**b-c**).

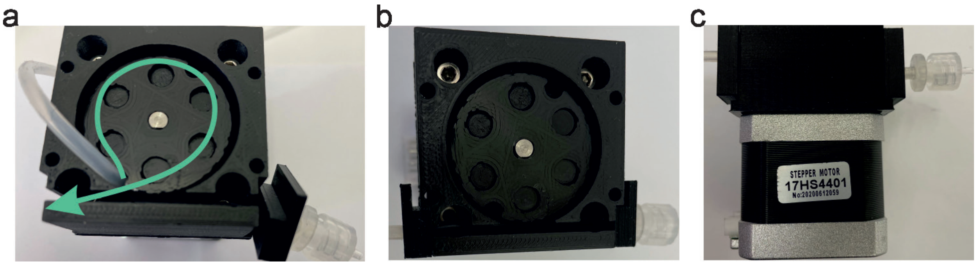

3. Construction guide for 3D-printed syringe pumps

3.1 Individual parts of syringe pump. All 3D printing files can be downloaded from GitHub https://github.com/KaetzelLab/Operant-Box-Design-Files/tree/main/3D%20Printed%20Syringe%20Pumps.

The syringe pump volume depends on the combination of stepper step size and spindle thread, low step size and small thread outputs lower the volume. We used 0.4 mm resolution and polylactic acids (PLA, biodegradable plastic) to print all 3D files parts.

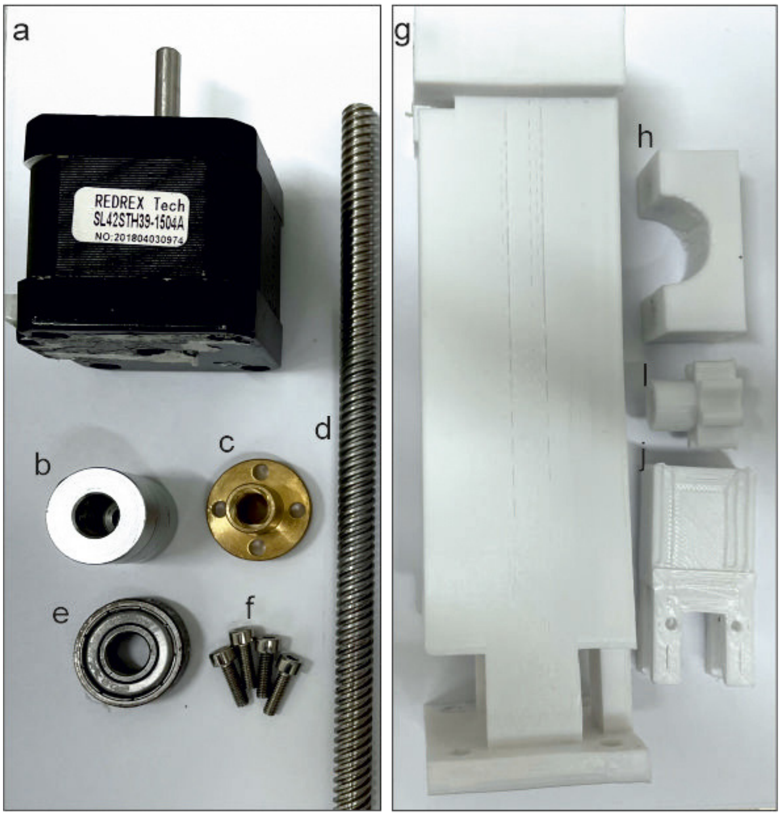

Parts shown in the figure (identified by letters), with quantity of each part per pump and design file for 3D-printing (.stl) or description (all non-printed items were obtained from Amazon):

Item, description	Q'ty	stl file / part name; source
a, stepper motor	1	Nema-17 stepper motor; e.g. Amazon (17HS4401)
b, motor-spindle coupler	1	flexible shaft coupling 5mm, aluminium; e.g. Yotino (Amazon)
c, d, spindle and nut	1	T8 trapeze guide thread spindle & nut, pitch 2mm, screw lead 8mm; e.g. Diyeeni (via Amazon)
e, metal ball bearing	1	Double shielded miniature groove ball bearings, 8x22x7 mm; e.g. HonLena 608 ZZ (Amazon)
f, screws	4	M3*6 mm screws with hex socket head, e.g. Farnell (1419600)
g , syringe holder	1	SPcap_v1.stl
h, housing structure	1	SyringePump(150mm)_V1.stl
i , knob to move spindle	1	SyringePumpKnob_v1.stl
j, syringe piston peddler	1	SyringePusher_v1.stl
(not shown), magnets	4	neodymium magnets, Ø 8 mm x 3 mm thick; e.g. Amazon

3.2 Mount **stepper motor** to main housing structure using 4 M3*6mm screws (**a**), and **T8 threaded spindle** to nut (**b**). Fix the **ball bearing** into the front end hole of the housing (**c**). Add 2 magnets to front top platform (**d**; use adhesive if necessary).

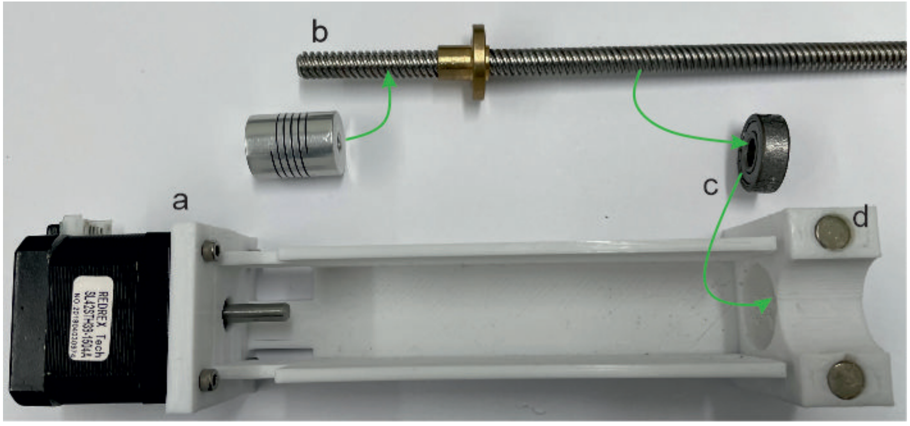

3.3 Couple **spindle** - passed through main house **ball bearing** - to stepper shaft using the **coupler**; slowly tight the screws on the coupler. Add 2 **magnets** to **cap** (use adhesive), and mount the **knob** to front end of the spindle.

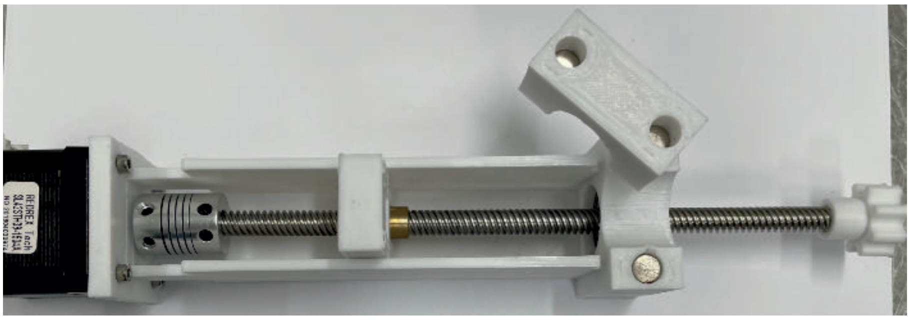

4. Installation guide for connecting and using pyOS-5 boxes

4.1 Install **Python3** from https://www.python.org/downloads/ and the following 5 packages using the respective pip command prompt: **numpy** , **pandas** , **pyserial** , **pyqtgrapgh** , **PyQt5.** Alternatively, install Python3 using Anaconda from https://docs.anaconda.com/anaconda/install and the required 5 packages using Conda. The figure shows example sceen shots of this process: (**a**) Opening terminal in Windows to install packages. (**b-c**) Installing packages using pip (package installer for python).

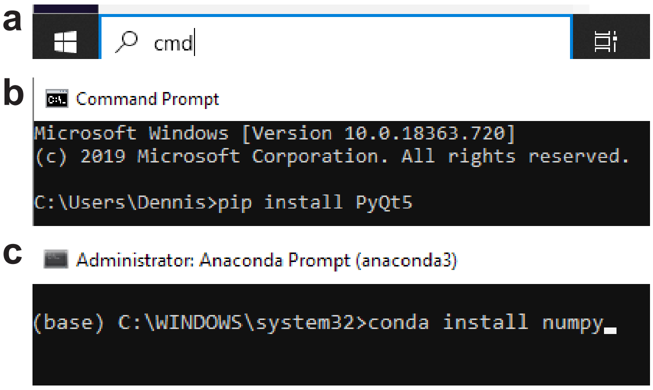

4.2 Go to the **GitHub** page https://github.com/KaetzelLab/Operant-Box-Code , click "Code" and then "Download ZIP" to **download the complete pyOS-5 code** as zipped folder to the controlling PC. Unzip the folder to obtain the following folder structure:

**Main folder**	**Files or subfolder**	**Description**
gui	GUI_Final.ui	Qt editable Graphical User interface template
	Run_5CSRTT.py	GUI for 5-choice serial reaction time task (5CSRTT)
	Run_Ruleshift.py	GUI for rule-shift task
	Run_DNMTP.py	GUI for delayed-non-matche-to-position (2CSWM) task
	Run_DMTP.py	GUI for delayed-matching-to-position (5CSWM) task
	Run_CPT.py	GUI for continuous performance task (CPT; Go/NoGo)
	port_list.py	Pre-assigned serial port numbers
com	data logger and pyboard serial communication	Serial communication and data logging
config	hardware configuration file	Configuration files
data	(as required)	Behavioural data
devices	input and output devices	pyControl hardware classes (uploaded to pyboard)
pyControl	framework files	pyControl framework (uploaded to pyboard)
tasks	5CSRTT	Task protocol file for 5-CSRTT
	Ruleshift	Task protocol file for rule-shift task task
	DNMTP	Task protocol file for DNMTP 2CSWM task
	DMTP	Task protocol file for DMTP 5CSWM task
	CPT	GUI for CPT

4.3 Upon the first installation, once all **peripherals** are connected as shown in Fig. 1 of the main manuscript, connect the pyControl breakout boards of all boxes *first* to their plugged-in **power supply** and *then*, *one-by-one* to the computer (via USB2.0 hub if >4 boxes are used), while observing the "Ports (COM/LPT)" list in the Windows "Device Manager". When a certain pyControl board is connected via USB, the number of the COM-port it gets assigned permanently by the PC will be shown in the Ports list (e.g. com1) - note down that number into the first section of the "**port_list.py" file** that defines the respective 8-box set-up (circled in the right figure; the remainder of the file can be kept as is unless another setup, e.g. 'Setup_2' is defined, requiring the respective additional definition with the corresponding change of name).

```
def select_setup_1(self):
    self.comboBox_srport_1.setCurrentText('com36')
    self.comboBox_srport_2.setCurrentText('com37')
    self.comboBox_srport_3.setCurrentText('com38')
    self.comboBox_srport_4.setCurrentText('com7')
    self.comboBox_srport_5.setCurrentText('com8')
    self.comboBox_srport_6.setCurrentText('com9')
    self.comboBox_srport_7.setCurrentText('com10')
    self.comboBox_srport_8.setCurrentText('com11')

    self.pushButton_setupselect_1.setStyleSheet('Setup_1')
    self.pushButton_setupselect_1.setText('SetUp1 : 01 - 08')
    self.pushButton_setupselect_2.setStyleSheet('Setup_2')
    self.pushButton_setupselect_3.setStyleSheet('Setup_3')
    self.pushButton_setupselect_1.setEnabled(False)
    self.pushButton_setupselect_2.setEnabled(False)
    self.pushButton_setupselect_3.setEnabled(False)
    self.pushButton_setupselect_2.setText(' ')
    self.pushButton_setupselect_3.setText(' ')
    self.label_box1.setText('Box 01')
    self.label_box2.setText('Box 02')
    self.label_box3.setText('Box 03')
    self.label_box4.setText('Box 04')
    self.label_box5.setText('Box 05')
    self.label_box6.setText('Box 06')
    self.label_box7.setText('Box 07')
    self.label_box8.setText('Box 08')
```

This file **assigns a permanent com serial number** to individual boards. One **port_list.py** file can store and thereby define up to 24 com ports (i.e. boxes) distributed over up to thre 8-box set-ups. Even though com ports can also be specified later in the GUI (see 4.4), for multiple boxes it is more convenient to define com numbers via the **port_list.py** file in the specific box order. The correct COM will then automatically be called when selecting the setup during the daily operation in the top-right corner of the main GUI window (see 4.5-4.6).
Note that the **GUI** also allows to select the **serial ports** for all boxes of an 8-box set-up at once or for each indivdual set-up ((h-i) in the screenshot in 4.5) and to connect all ports at once by clicking **Dis/Con** or to connect individual boxes by clicking **Connect** - if they are not pre-assigned in the 'port_list.py' file as just described.

4.4 Once at the beginning of an experimental series with a certain task-type (e.g. 5CSRTT), define the task-type and its hardware framework (determining inputs and outputs connected to the pyControl breakout board). To do this, simply go into the "tasks" folder and then the subfolder of the chosen task, copy all specific files in there, go two levels up again to the main task folder and paste the copied task files there. All specific task files in this main folder are those that will be visible and **selectable in the "TASK" drop-down menu of the GUI later**. Note that the provided individual task files will not only contain task-specific files but also auxillary programmes, e.g. for the prior habituation training, for checking all poke-holes at the beginning of the daily runs, and for running the pump to load reward or clean the tube.
If multiple different tasks are run on the same day (e.g. 5CSRTT and 5CSMWT), it is best to create two different main folders, one for each task and with the respective task-specific files copied into the top-level folder, which may be named by the task name (e.g., 5CSRTT, see below).

To **define the hardware framework** for a given set-up and task, open the **GUI** (as described in 4.5) and click "**Config All**" (or "Config", in case a hardware definition should be made only for specific boxes; (j) in the screenshot below). This action evokes a window providing the option to select and upload a **framework file** and a **hardware definition file** from the *config* folder.
(**1**) The **framework file** defines how pyControl handles events, inputs and output and would usually not be changed by the user; therefore the path is pre-set to the default framework file which is uploaded upon pressing "load framework".
(2) The **hardware definition file** defines the input and output peripherals and their basic properties, and therefore relates to the specific wiring between the *pyControl breakout board* and the *peripherals* that are connected to it. For the typical pyOS5 setup described in the main manuscript (Fig. 1), select the file **pyOS5_HardwareDefinition.py**. The configuration file may change, if additional inputs/output for integration with physiological recordings or optogenetic stimulation are defined (see accompanying main manuscript, Fig. 7b); such extended hardware definition files are also provided in the *config* folder and can be selected by clicking "Load hardware definition".

4.5 Daily operation 1: Start the GUI for the desired task (5CSRTT in the example below). This can be done in different ways: Either call the task-specific GUI name in the Windows Command prompt; to do this go to the respective GUI-folder in Windows Explorer and type "cmd" in the address line at the top, then type "python [GUI-name.py]", e.g. "python Run_5CSRTT.py"; see screenshot. Alternatively, if python is added to the path during installation, one can open the GUI simply by double-clicking on the task-file.

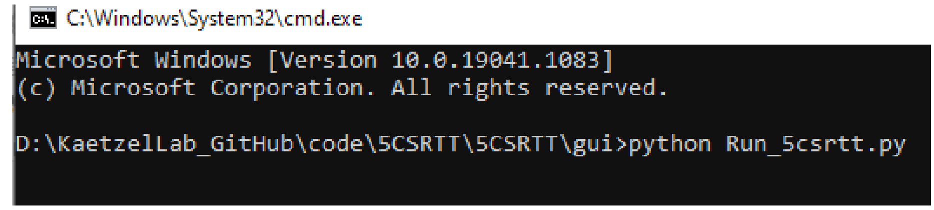

The opened **GUI** has the following elements as shown in this screen shot of its Main tab: (**a**) Data directory selection; (**b**) Selection of setup to assign serial port number saved in port_list.py (the GUI assumes 3 set-ups of 8 boxes each, of which the one to be used has to be chosen; (**c - f**) fields for drop-down menu-based selection (c), upload (d-e) and start (f) of a specific task file; (**g**) buttons to export data after an experiment; (**h-j**) fields for assigning com port number (i.e. individual box; h), and connecting to that port (i) and configuring the hardware framework (j).

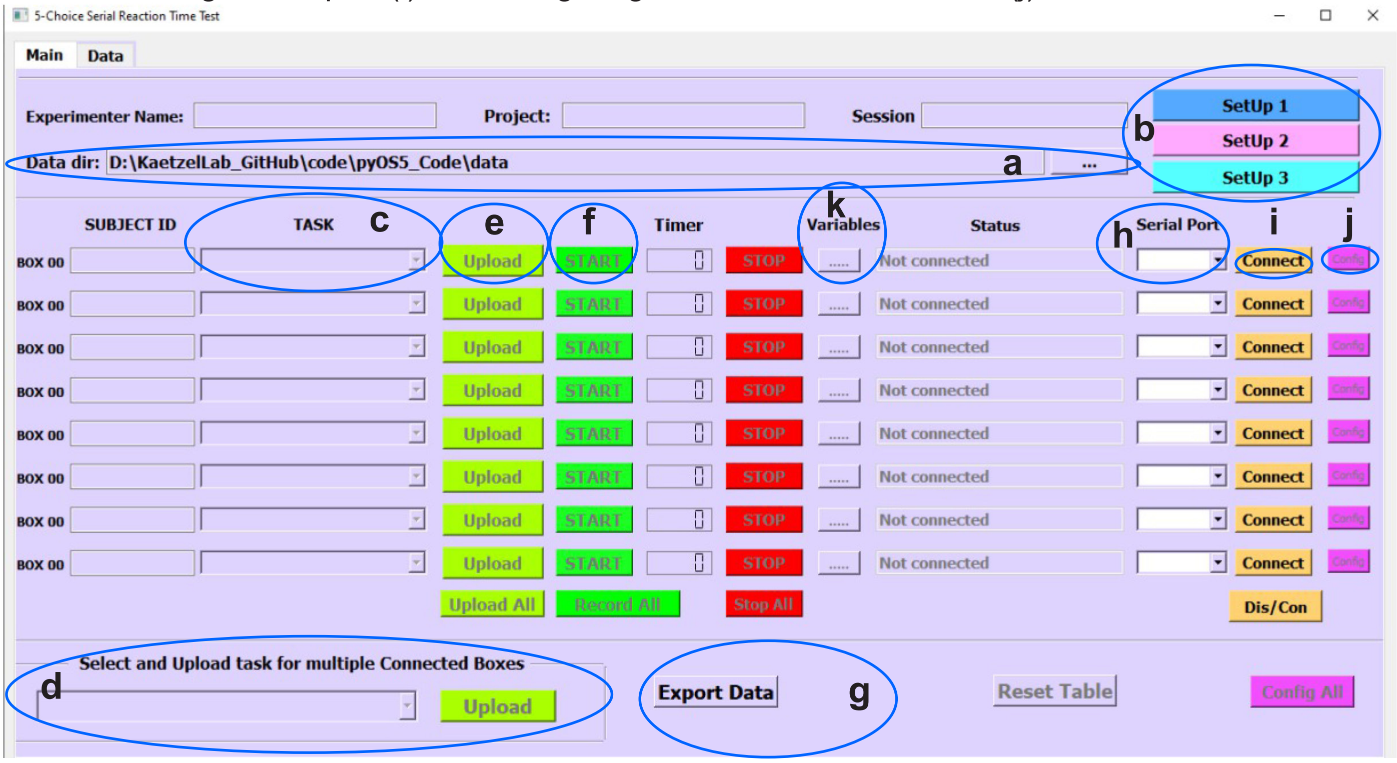

4.6. Daily operation 2: Set-up and start the experiment.
(**1**) Select the setup (clicking the respective button, b)
(**2**) Select a **task file** from the **drop-down menu** (c/d) and press **upload** (e/d)**.** The user can upload different task files to different boxes in the same GUI *individually* or can upload the *same* task file to *all* connected boxes from the *common drop-down menu* located at the bottom left of the GUI (d). Note that one can also change individual task parameters from the GUI by clicking the **Variables** button (k; this is usually not necessary, because all parameters are specified in the task file).
(**3**) Enter a **subject id** to store and save individual csv files (containing all events and timestamps) for the respective animal. Optionally, experiment name, project and session can also be added and the location where the data is saved can be changed (a). If you entered subject id, the **Start** button (f) will turn to **Record**.
(**4**) Press **Record** to start the experiment.

4.7 Daily operation 3: Conclusion of the experiment. For each experiment, **two types of results files** will be created:
(**1**) one that records the **aggregated values** (e.g. number correct responses, accuracy, etc.) for **all animals** in the 8-box set-up at once (as seen continuously in the “Data” tab of the GUI). To obtain this results file, press “Export Data” (g) and later reset the data table by clicking “Reset Table”. (The “Reset Table” button will not work before the data has been exported in order to prevent accidental clearing of data.)
(**2**) Additionally, one file will be created for each subject (identified by the subject ID specified in 4.6.3) that stores a **record of all behavioural events** with millisecond precision time-stamps. This file is already created automatically during the experiment.
